# Performance of piezoelectric beam type energy harvester under flow-induced vibration

**DOI:** 10.1038/s41598-025-98147-0

**Published:** 2025-04-18

**Authors:** Amya Ranjan Ray, Santanu Koley

**Affiliations:** Department of Mathematics, Birla Institute of Technology and Science - Pilani, Hyderabad Campus, Telangana, 500078 India

**Keywords:** Piezoelectric, PZT-5A, ANSYS-Fluent, Turbulent flow, Flow-induced vibration, Engineering, Mechanical engineering

## Abstract

The objective of this research is to study the hydrodynamics and performance of piezoelectric PZT-5A patches in the context of turbulent fluid flow, with a specific focus on the impact of structural positioning on output performance. The numerical three-dimensional model consists of piezoelectric PZT-5A patches affixed to beams and positioned inside a uniform circular pipe. The modeling and simulation of the piezo-beams are conducted using ANSYS-Fluent software to analyze the vortex shedding phenomenon, which occurs due to induced vibrations from the interaction of the fluid with the PZT beams. A vorticity analysis is included to better understand the flow dynamics around the structures, revealing insights into the flow patterns that enhance energy harvesting. Additionally, a grid independence study is performed to ensure that the numerical results are reliable and not dependent on the grid resolution. This study confirmed that the results are consistent across different mesh densities, providing confidence in the accuracy of the simulations. The study also explores how turbulent flow increases the output voltage. It examines the effect of varying the spacing distance between two structures, considering three cases with distances of $d_2=0.0505$ m, 0.101 m, and 0.202 m, to evaluate their influence on natural frequencies and voltage output. The results indicate that the spacing significantly affects energy transfer and voltage generation, with the optimal configuration observed at $d_2=0.202$ m. The maximum voltage value of 0.41264 V is observed from the structures across a broad frequency range. This voltage can be utilized in micro-scale applications such as cell phone charging, glowing LED lights, and bulbs, and can also be stored in batteries for future use.

## Introduction

With the fast expansion of the global economy, energy demands are increasing rapidly, particularly in countries with emerging economies. However, non-renewable energy sources are unable to fulfill these demands due to their scarcity and limited availability in nature, as well as their detrimental effects on the environment. To fulfill the substantial energy demand, renewable energy sources are being increasingly considered. It has tremendous potential and has the ability to reduce global energy consumption in general, which is derived predominantly from fossil fuels and attenuates the release of greenhouse gases into the atmosphere^1^. It is abundant in nature and easily accessible to mankind around the world. Migrating to renewable energy sources for electricity generation is economically advantageous and beneficial to the healthy environment^2,3^.

To meet the goal of creating sustainable and environmentally friendly energy solutions, energy harvesting has gained popularity in recent years as an innovative approach. Mahapatra et al.^4^ elucidated the conversion of mechanical energy, such as vibration energy, into electrical energy to offer a cleaner alternative to conventional methods such as solar panels and nuclear energy, which often entail environmental drawbacks. Concurrently, Lu et al.^5^ explored nonlinear energy harvesting from fluid-conveying piezoelectric pipes, emphasizing the importance of efficient energy conversion with minimal environmental impact. Their study not only showcases the potential for sustainable energy generation but also underscores the necessity of eco-friendly technologies to combat environmental degradation. Building on this foundation, Hafizh et al.^6^ developed a non-linear airfoil-shaped piezoelectric energy harvester designed to convert the flow-induced vibrations from water into electrical energy for IoT applications. Through the utilization of renewable energy sources and the reduction of reliance on fossil fuels, these technologies play a crucial role in mitigating greenhouse gas emissions and fostering environmental cleanliness. Furthermore, the inclusion of a passive self-adjustable base in the design demonstrates a commitment to energy efficiency and resource conservation, further enhancing the environmental sustainability of the device. Similarly, Dhingra et al.^7^ highlighted the potential of piezoelectric materials in providing cleaner energy solutions, particularly in powering lighting systems and other equipment. Through innovative research and technological developments, piezoelectric energy harvesting offers a pathway towards achieving a greener and more sustainable future, in which environmental considerations are prioritized alongside the energy generation efficiency.

The discovery of the piezoelectric effect by Jacques and Pierre Curie in 1880 marked a pivotal moment in energy harvesting research^8^. This effect, which enables certain materials to generate electric charges in response to mechanical stresses or vibrations, forms the cornerstone of various energy conversion methods aimed at sustainability. Significantly, vortex-induced vibration (VIV) stands out as a technique harnessing fluid energy and bluff bodies to induce vibrations in piezoelectric energy harvesters (PEHs), thereby converting mechanical energy into electrical power. Fang^9^ advanced this field by developing a micro piezoelectric power generator employing a composite cantilever structure and PZT material. Fabricated with micro-electromechanical system (MEMS) techniques, the device made of PZT material effectively harnesses ambient vibration energy through the piezoelectric effect, resonating in low-frequency environmental vibrations. Gamayel et al.^10^ expanded on this concept, providing experimental and simulation results that underscored the efficacy of VIV-based energy harvesting, particularly in different airflow velocities. Laws et al.^11^ enhanced energy harvesting efficiency by incorporating Y-shaped fins onto a square bluff body and employed computational modeling to investigate the impact of fin addition on steady-state amplitudes. Wang et al.^12^ investigated the vortex-induced vibration response of bluff bodies with various rear-end shapes (semicircular, triangular, trapezoidal, and rectangular) using computational fluid dynamics (CFD) methods. Furthermore, Covaci and Gontean^13^ conducted a comprehensive review of energy harvesting methods, emphasizing advancements in piezoelectric systems that enhance energy output in fluid flow environments. By integrating these advanced techniques, significant strides have been made toward achieving more sustainable and environmentally friendly energy solutions, highlighting the pivotal role of piezoelectric technology in addressing contemporary environmental challenges.

In the realm of micro-level power generation, flow-induced vibration emerges as a promising avenue for harnessing energy from fluid dynamics. Du et al.^14^ introduced the concept of capturing mechanical oscillations induced by fluid flow and converting them into electrical energy using piezoelectric materials. This approach holds significant potential for providing sustainable energy solutions, particularly in environments with consistent fluid flow patterns. Li et al.^15^ further explored this concept by investigating a novel energy harvesting structure based on the flow-induced piezoelectric vibration, optimizing power output by aligning resonant frequencies with vortex-shedding frequencies through additional patches. Tang et al.^16^ contributed to the theoretical foundation of microscale power generation systems by developing a comprehensive model for flexural vibrations of microbeams in flow, highlighting the intricate dynamics involved in energy extraction. Zhu et al.^17^ investigated flow-induced vibration of trapezoidal cylinders, observing VIV-desynchronization and galloping phenomena. They examined energy transfer characteristics and the influence of cylinder shape on vortex shedding modes. Amiri et al.^18^ delved into the instability and vibrations of fluid-conveying piezoelectric nanotubes, offering insights crucial for the efficient design of such systems. Together, these studies emphasize the potential of flow-induced vibration as a sustainable energy source, with implications for renewable energy development and microscale power generation.

In pursuing sustainable energy solutions, leveraging flow-induced vibrations for energy harvesting emerges as a significant stride. Integrating piezoelectric transducers into structures exposed to fluid flow not only taps into renewable energy sources but also advocates for environmental conservation. Wu et al.^19^ explored the high-power output from piezoelectric mechanisms in structures by focusing on flow-induced vibrations. The study highlighted the energy harvesting methods from ambient vibrations and the potential of additive manufacturing for creating efficient piezoelectric materials tailored for FIV applications. Borisov et al.^20^ examined the porous micro-beams equipped with two piezoelectric layers to convert the fluid flow-induced vibrations into electricity. Lee et al.^21^ investigated piezoelectric flow energy harvesting for deep oil well applications using a cantilever beam configuration. Additionally, the study explored two electromechanical coupling designs: a piezoelectric bimorph cantilever and a cantilever mounted on flex-tensional actuators. Experimental results showed that both designs generate up to 20 mW of power at a flow rate of 20 L/min. The capacities and efficiencies of four micro-power harvesting techniques: thermoelectric, thermo-photovoltaic, piezoelectric, and microbial fuel cell renewable power generators were examined by Selvan and Ali^22^. Additionally, this work discussed the challenges, benefits, applications, and constraints of each method. The aforementioned innovative configurations offer advantages in bolstering energy efficiency while mitigating environmental impact. Delving into diverse structural designs and PZT configurations extends beyond optimizing energy generation to aligning efforts with environmental sustainability objectives. This research aims to advance this cause by exploring methodologies that maximize energy output while upholding environmental integrity, contributing to a cleaner and greener future.

In this paper, the micro-level analysis is conducted to explore electricity generation via piezoelectric structures, focusing on the utilization of two beam structures with PZT patches placed inside a pipe conveying fluid. While previous researches were primarily focused on single PZT beam configurations or various structural arrangements, this study addresses the gap by investigating the behavior of multiple beam structures with three distinct configurations ($d_2=0.0505$ m, 0.101 m, and 0.202 m) in a turbulent flow environment. By analyzing the hydrodynamics of the piezoelectric energy harvesters, vibrational properties, and mechanical behavior of the rectangular structures within the context of turbulent flow, the study aims to provide insights into the performance and efficiency of this energy harvesting approach. The organization of the paper includes detailed modeling of the three-dimensional cylindrical pipe with circular cross-section, geometry, grid generation, governing equations, and associated boundary conditions provided in Sect. 2. Section 3 delves into the hydrodynamics of the PEHs, vibrational properties, and mechanical behavior of the rectangular structures. The results and conclusions derived from the research findings are presented in Sects. 4 and 5, respectively, highlighting the significance of the study in advancing the understanding of micro-scale energy harvesting in turbulent flow environments.

## Mathematical formulation

This section contains the mathematical modeling of the Piezo-beam structure energy harvester inside a right circular cylindrical pipe in the Cartesian coordinate system in three dimensions. In this particular case, the *z*-axis is oriented in the lateral direction, the *y*-axis is taken in the vertical direction, and the *x*-axis is aligned along the horizontal direction. The present analysis is conducted using a cylindrical structure to consider the numerical simulations. The cylindrical three-dimensional CFD model is generated in ANSYS 2022 R1 (https://www.ansys.com/) software.

**Figure 1:**
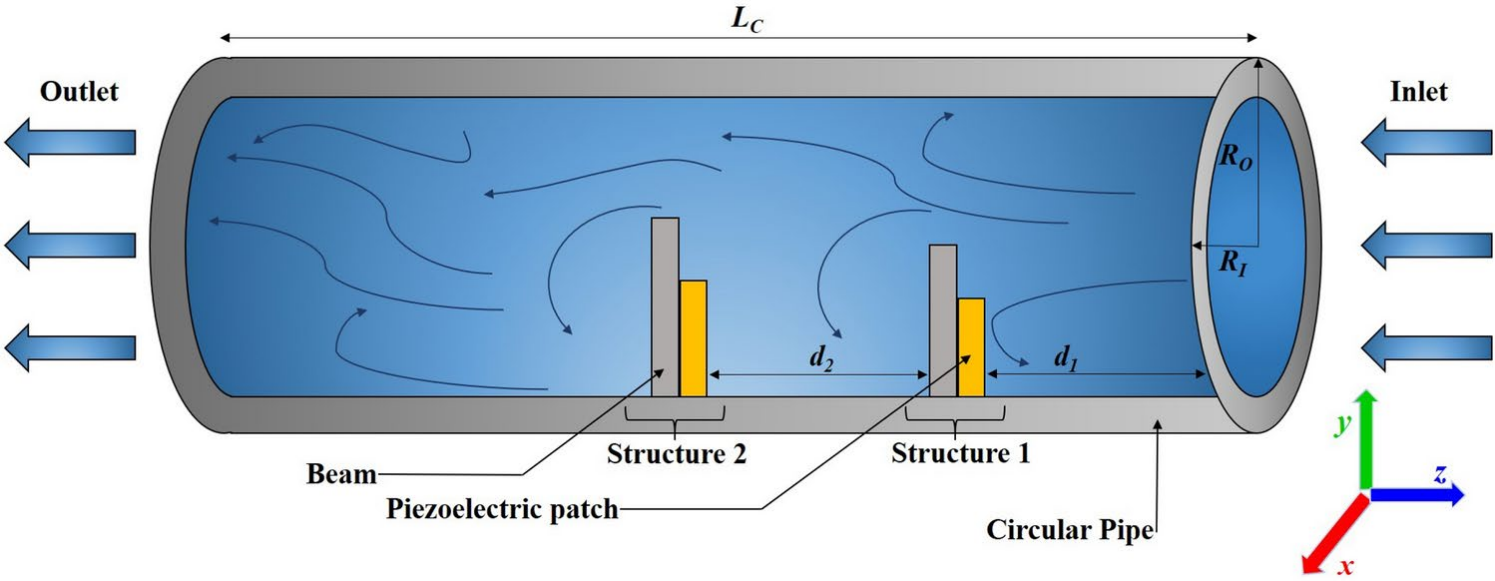
Schematic view of the flow-based piezoelectric energy harvester.

### Designing and modeling of piezoelectric energy harvesters

In the present work, a right circular cylindrical pipe is the computational domain of the ongoing problem, along with a piezoelectric patch attached to the vertical beam constructed inside it by utilizing ANSYS 2022 R1 software. The circular pipe that makes up the planned energy harvesting prototype has the following measurements: 1.0 m in length, 0.20 m in outer diameter, 0.18 m in inner diameter, and 0.01 m in thickness. Two rectangular beams having dimensions of 0.04 m $\times$ 0.02 m $\times$ 0.02 m and 0.03 m $\times$ 0.02 m $\times$ 0.02 m are mounted inside the circular pipe. PZT-5A piezo patches have been used, which have a size of 0.03 m $\times$ 0.02 m $\times$ 0.02 m and 0.02 m $\times$ 0.02 m $\times$ 0.02 m at a distance $d_1=0.202$ m from an inlet boundary, as shown in Fig. 1. The configuration that is being shown aims to use a piezo patch to convert the hydro-kinetic energy of a fluid flowing at a rate of 1.00 m/s into electricity. This case involves a spacing distance of $d_2=0.202$ m between structure 1 and structure 2. The model’s physical measurements and configurations are listed in Table 1.

**Table 1:** Dimensions and physical properties of the appropriate circular cylindrical construction.

Symbol	Meaning	Values	Unit
$R_O$	Outer radius of cylinder	0.10	m
$R_I$	Inner radius of cylinder	0.09	m
$T_C$	Thickness of the cylinder	0.01	m
$L_C$	Length of the cylinder	1.00	m
$\nu$	Kinematic viscosity	$1\times 10^{-6}$	$\hbox {m}^2$/s
$l_{B1}$	Length of the first beam	0.03	m
$l_{B2}$	Length of the second beam	0.04	m
$W_B$	Width of the beams	0.02	m
$T_B$	Thickness of the beams	0.02	m
$L_{PZT1}$	Length of the first PZT piezo-patch	0.02	m
$L_{PZT2}$	Length of the second PZT piezo-patch	0.03	m
$B_{PZT}$	Breadth of the PZT Piezo-patches	0.02	m
$h_{PZT}$	Thickness of the PZT piezo-patches	0.02	m
$U_0$	Fluid flowing velocity at inlet	1.00	m/s
$\rho$	Fluid density	997.77	kg/$\hbox {m}^3$

#### Material selection

For this study, structural steel and PZT-5A are selected to meet the structural and energy harvesting requirements inherent to the system’s design. Structural steel is chosen as the structural material due to its high strength, corrosion resistance, and suitability for withstanding under fluid-induced stresses, ensuring durability and reliability. PZT-5A, a well-regarded piezoelectric ceramic, is selected for its high piezoelectric coefficient, enabling effective conversion of mechanical energy from vibrations into electrical output. The engineering data for both structural steel and PZT-5A are detailed in Table 2 for accurate simulation input. To ensure compatibility with ANSYS Workbench, the material properties presented in Table 2 are adapted to align with the software’s requirements. Conversion matrices, illustrated in Table 3, facilitate this adaptation by enabling precise input of the anisotropic elasticity, relative permittivity, and piezoelectric constants for PZT-5A. Table 3 represents the symmetric diagonal matrices for anisotropic elasticity and anisotropic relative permittivity. These matrices are provided as tabular data, allowing ANSYS to accurately capture the material’s directional characteristics and complex interactions under operational loading conditions.

**Table 2:** Engineering material properties.

Material	Properties	Value
Structural steel	Density $\left( \text {kg/m}^{3}\right)$	7850
	Poisson’s ratio	0.3
	Young’s modulus (GPa)	200
PZT-5A	Density (kg/$\hbox {m}^{3}$)	7750
	Anisotropic elasticity	
	Stiffness matrix (GPa)	
	$D_{11}=D_{22}$	121
	$D_{12}$	75.2
	$D_{13}=D_{23}$	75.1
	$D_{33}$	111
	$D_{44}$	22.6
	$D_{55}=D_{66}$	21.1
	Piezoelectric matrix $\left( \text {C/m}^{2}\right)$	
	$e_{13}=e_{23}$	-5.4
	$e_{33}$	15.8
	$e_{61}=e_{52}$	12.3
	Anisotropic relative permittivity	
	Dielectric matrix at constant strain (nF/m)	
	$\epsilon ^S_{11}=\epsilon ^S_{22}$	1.99
	$\epsilon ^S_{33}$	5.78

### Meshing

**Figure 2:**
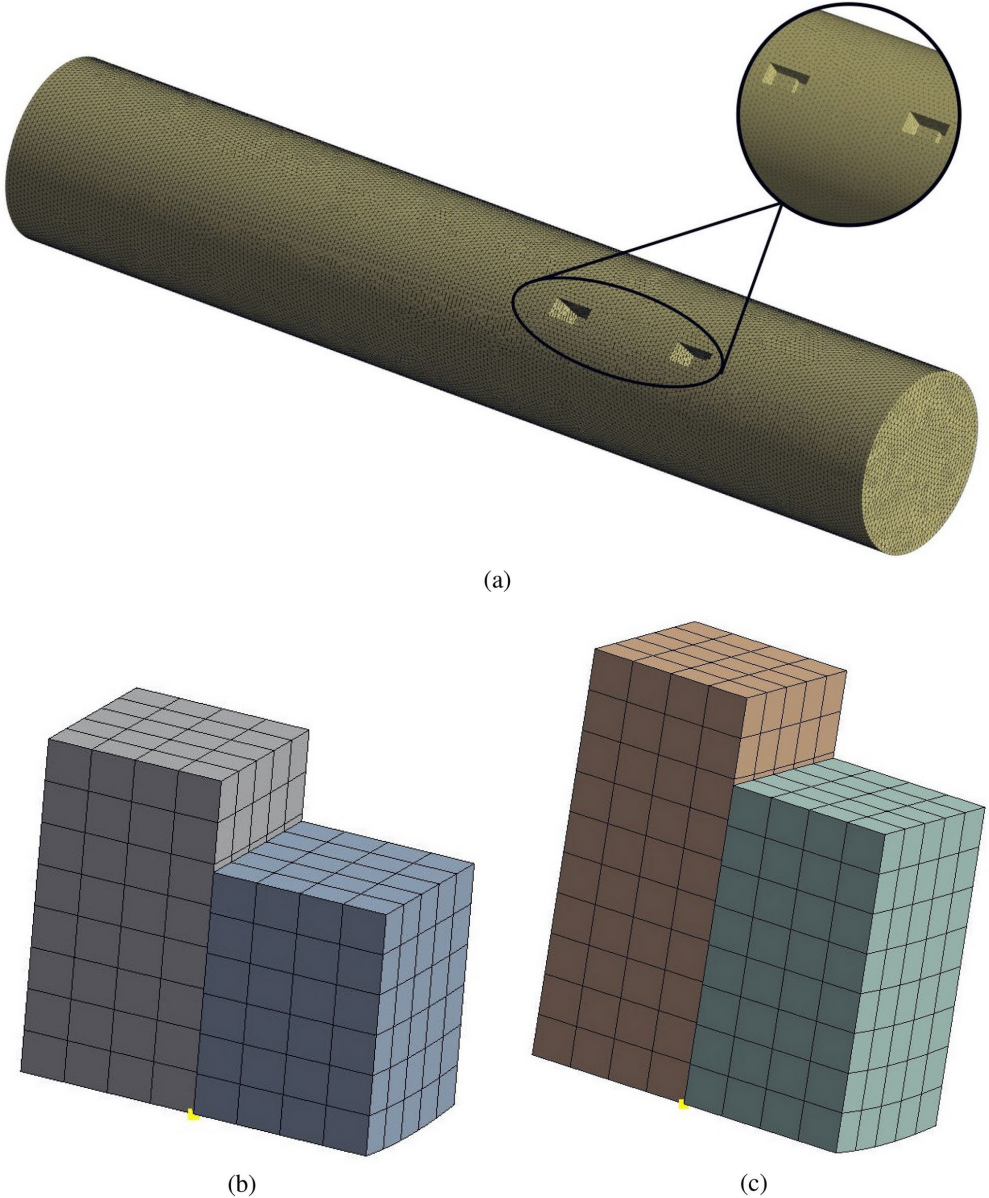
Descritization of the CFD domain, (**a**) circular pipe, (**b**) structure 1, and (**c**) structure 2, using tetrahedral and hexahedral elements.

An effective meshing process is pivotal to the success of CFD simulations, as it ensures the accurate representation of complex geometries and flow phenomena. In this study, ANSYS meshing is employed to develop an optimized mesh configuration, as illustrated in Fig. 2. The pipe is discretized using tetrahedral mesh elements with an element size of 0.005 m, providing the necessary flexibility to capture the intricate flow dynamics. This results in a total of 1, 740, 047 elements and 306120 nodes for the circular pipe. Conversely, the piezo-beam structures are meshed with hexahedral elements, using an element size of 0.005 m.

The quality of the meshing used in the CFD model is further validated by key mesh metrics: the average element quality is 0.993, skewness is 0.018, aspect ratio is 1.049, and orthogonal quality is 0.798. This strategic combination of tetrahedral and hexahedral meshing guarantees good mesh quality, which is essential for obtaining reliable simulation results. Through careful adjustment of mesh parameters, the model achieves a balance between the computational efficiency and the accuracy required for effective analysis.

**Table 3:** Piezoelectric material parameters of the PZT-5A.

*D*: anisotropic elasticity (Pa)					
$1.2 \times 10^{11}$	$7.52 \times 10^{10}$	$7.51 \times 10^{10}$	0	0	0
$7.52 \times 10^{10}$	$1.2 \times 10^{11}$	$7.51 \times 10^{10}$	0	0	0
$7.51 \times 10^{10}$	$7.51 \times 10^{10}$	$1.11 \times 10^{11}$	0	0	0
0	0	0	$2.26 \times 10^{10}$	0	0
0	0	0	0	$2.11 \times 10^{10}$	0
0	0	0	0	0	$2.11 \times 10^{10}$

*e*: piezoelectric matrix (C/$\hbox {m}^2$)					
0	0	$-5.4$			
0	0	$-5.4$			
0	0	15.8			
0	0	0			
0	12.3	0			
12.3	0	0			

$\epsilon ^S$: anisotropic relative permittivity					
$1.99 \times 10^{-9}$	0	$-5.4$			
0	$1.99 \times 10^{-9}$	$-5.4$			
0	0	$5.78 \times 10^{-10}$			

### Governing equations

In this study, the governing equations for fluid motion are rooted in principles of mass and momentum conservation and are crucial to understand the complex systems. The continuity equation for an incompressible flow is given as

1 $$\begin{aligned} \displaystyle \rho (\nabla .\varvec{u})=0, \end{aligned}$$

where $\rho$ is the density of the fluid, assumed constant for incompressible flows and $\varvec{u}$ is the velocity field of the fluid. This equation expresses the conservation of fluid mass, quantifying the rate of change within a specified region. Simultaneously, the conservation of momentum

2 $$\begin{aligned} \displaystyle \rho (\varvec{u}.\nabla )\varvec{u}=\nabla .[-pI+\varvec{k}]+\varvec{F}, \end{aligned}$$

provides insights into the forces driving fluid motion, contributing to a comprehensive framework. Here, $\rho$ represents the fluid density, $\varvec{u}$ is the velocity vector, *p* is pressure, *I* is identity matrix and $\varvec{F}$ denotes external body forces per unit mass (see Luo et al.^23^).

### $k-\epsilon$ model

In incompressible steady flows simulated using Reynolds-Averaged Navier-Stokes (RANS) modeling, turbulence is characterized by a turbulent viscosity hypothesis model. This model, as investigated by Verma and Sonparote^24^, plays a crucial role in representing the effects of unresolved turbulent fluctuations. In the current investigation, the $k-\epsilon$ model is used to examine the impact of turbulent flow. In addition, the governing equations are provided as follows

3 $$\begin{aligned} \displaystyle \rho (\varvec{u}.\nabla )k= & \nabla .[(\mu +\frac{\mu _T}{\sigma _K})\nabla k]+P_k-\rho \epsilon , \end{aligned}$$

4 $$\begin{aligned} \displaystyle \rho (\varvec{u}.\nabla ) \epsilon= & \nabla .[(\mu +\frac{\mu _T}{\sigma _\epsilon })\nabla \epsilon ]+C_{\epsilon _1}\frac{\epsilon }{k}P_k-C_{\epsilon _2}\rho \frac{\epsilon ^2}{k}. \end{aligned}$$

Here, Eqs. (3) and (4) represent the transport equation for TKE and turbulent dissipation rate, respectively. The equations for the production term and turbulent viscosity are given by

5 $$\begin{aligned} \displaystyle P_k= & -\rho \epsilon , \end{aligned}$$

6 $$\begin{aligned} \displaystyle \mu _T= & \rho C_\mu \frac{k^2}{\epsilon }. \end{aligned}$$

### Wall movement equations

In the simulation of turbulent flows, adherence to boundary conditions and specialized equations near solid walls are paramount for an accurate representation of fluid dynamics. The shear condition characterizing the turbulence layer at the fluid-solid interface in a region featuring steady wall motion and a no-slip condition is articulated through the following equations

7 $$\begin{aligned} & \displaystyle \varvec{u}.\hat{\varvec{n}}=0, \end{aligned}$$

8 $$\begin{aligned} & \displaystyle k\hat{\varvec{n}}=-\rho \frac{u_\tau }{u^+}\varvec{u}_{tang}, \end{aligned}$$

9 $$\begin{aligned} & \displaystyle \varvec{u}_{tang}=\varvec{u}-(\varvec{u}.\hat{\varvec{n})\hat{\varvec{n}}}, \end{aligned}$$

10 $$\begin{aligned} & \displaystyle \nabla k.\hat{\varvec{n}=0}, \end{aligned}$$

11 $$\begin{aligned} & \displaystyle \epsilon =\rho \frac{C_\mu k^2}{k_\nu {\delta ^+}_w \mu }\varvec{u}_{tang}\nu . \end{aligned}$$

Here, Eqs. (7) and (10) represent the no-slip and Neumann boundary conditions, respectively. Further, Eqs. (8), (9), and (11) represent TKE near the wall, the tangential component of the velocity, and the turbulent dissipation rate near the wall. For all other boundaries, the velocity $u^+$ is precisely determined at a defined distance from the solid surface. This constant value is strategically chosen within the fully turbulent region of the system, ensuring an accurate representation of the flow dynamics near these boundaries.

### Fluid inlet-flow equations

In the context of inlet flow conditions, a fundamental set of equations models turbulent flows near solid boundaries. The normal velocity components are defined by

12 $$\begin{aligned} \displaystyle \varvec{u}=-U_0\hat{\varvec{n}}, \end{aligned}$$

where $U_0$ represents a constant velocity, and $\hat{\varvec{n}}$ is the unit normal vector to the surface. This formulation captures the intricate velocity behavior near the solid boundary, providing essential insights into the initial conditions of the turbulent flow. Simultaneously, standard normalization of the velocity throughout the simulation is expressed as

13 $$\begin{aligned} \displaystyle U_{ref}=U_0. \end{aligned}$$

Turbulence characteristics are represented by the turbulent kinetic energy (*k*) equation as

14 $$\begin{aligned} \displaystyle k=\frac{3}{2}(U_{ref}I_T)^2, \end{aligned}$$

where, *k* is linked to the reference velocity ($U_{ref}$) and turbulence intensity ($I_T$). Similarly, the turbulent dissipation rate is expressed as

15 $$\begin{aligned} \displaystyle \epsilon ={C_\mu }^{3/4}\frac{k^{3/2}}{L_T}. \end{aligned}$$

Here, we have incorporated a turbulence model constant ($C_{\mu }$) and a turbulent length scale ($L_T$).

### Fluid discharge equations

The total pressure is calculated using a precision gauge in the absolute frame of reference. When considering backflow pressure in the direction perpendicular to the system’s boundary, the subsequent equations are given as

16 $$\begin{aligned} \displaystyle [-pI+\text {k}]\hat{\varvec{n}}=\widehat{p_0}\hat{\varvec{n}}. \end{aligned}$$

Here, k and $p_0$ are the Von Karman constant and the discharge flow pressure, respectively. These also satisfies the following

17 $$\begin{aligned} & \displaystyle \widehat{p_0}\le p_0,\end{aligned}$$

18 $$\begin{aligned} & \displaystyle p_0=0. \end{aligned}$$

Furthermore, to encapsulate the behavior and interactions near the outlet point, providing essential constraints that contribute to a comprehensive understanding of the fluid dynamics in that specific region, the governing equations take the form

19 $$\begin{aligned} & \displaystyle \nabla k.\hat{\varvec{n}}=0,\end{aligned}$$

20 $$\begin{aligned} & \displaystyle \nabla \epsilon .\hat{\varvec{n}}=0. \end{aligned}$$

### Structure vibration and oscillation

Understanding the vibration and oscillatory response of the structure to external forces is crucial to investigate the dynamic behavior of the above computational systems. Vortex flow vibrations emerge from the interaction between the fluid and the piezoelectric beam structure. In this research, the Reynolds number is a key parameter for evaluating the interaction of a circular pipe with vortices under steady-state flow conditions. The Reynolds number is given by

21 $$\begin{aligned} \displaystyle Re=\frac{2R_IU_S}{\nu _k}. \end{aligned}$$

Further, the emergence of vortex shedding frequency results from the dynamic oscillating circulation process when the fluid enters the pipe. This frequency is precisely defined using the Strouhal number (*St*), which characterizes the shedding frequency of vortices in fluid flows around the structures. The relationship between the Strouhal number and the vortex shedding frequency ($f_S$) is given as

22 $$\begin{aligned} \displaystyle St=\frac{2f_SR_I}{U_S}. \end{aligned}$$

### Piezoelectric equations

Mathematically, piezoelectricity is described by the constitutive equation of a material, detailing the relationships between the electric displacement ($\varvec{D_e}$), the strain exerted on the piezo-patch ($\varvec{\sigma _S}$), the electric field ($\varvec{E_f}$) and the stress produced in the structure ($\varvec{\delta _S}$). The piezoelectric constitutive law represented in the Stress-Charge form is given by

23 $$\begin{aligned} \displaystyle \varvec{D_e}= & \epsilon ^S \varvec{E_f}+e \varvec{\sigma _S},\end{aligned}$$

24 $$\begin{aligned} \displaystyle \varvec{\delta _S}= & D \varvec{\sigma _S}-e^T \varvec{E_f}. \end{aligned}$$

The coefficients $\epsilon ^S$, *e*, and *D* characterize the piezoelectric properties governing the material’s response to mechanical and electrical influences. The values of these properties are comprehensively detailed in Table 2. Further, their corresponding matrix forms are elegantly illustrated in Table 3, offering a clear representation of the PZT equations.

### Fluid-piezoelectric interaction modeling

**Figure 3:**
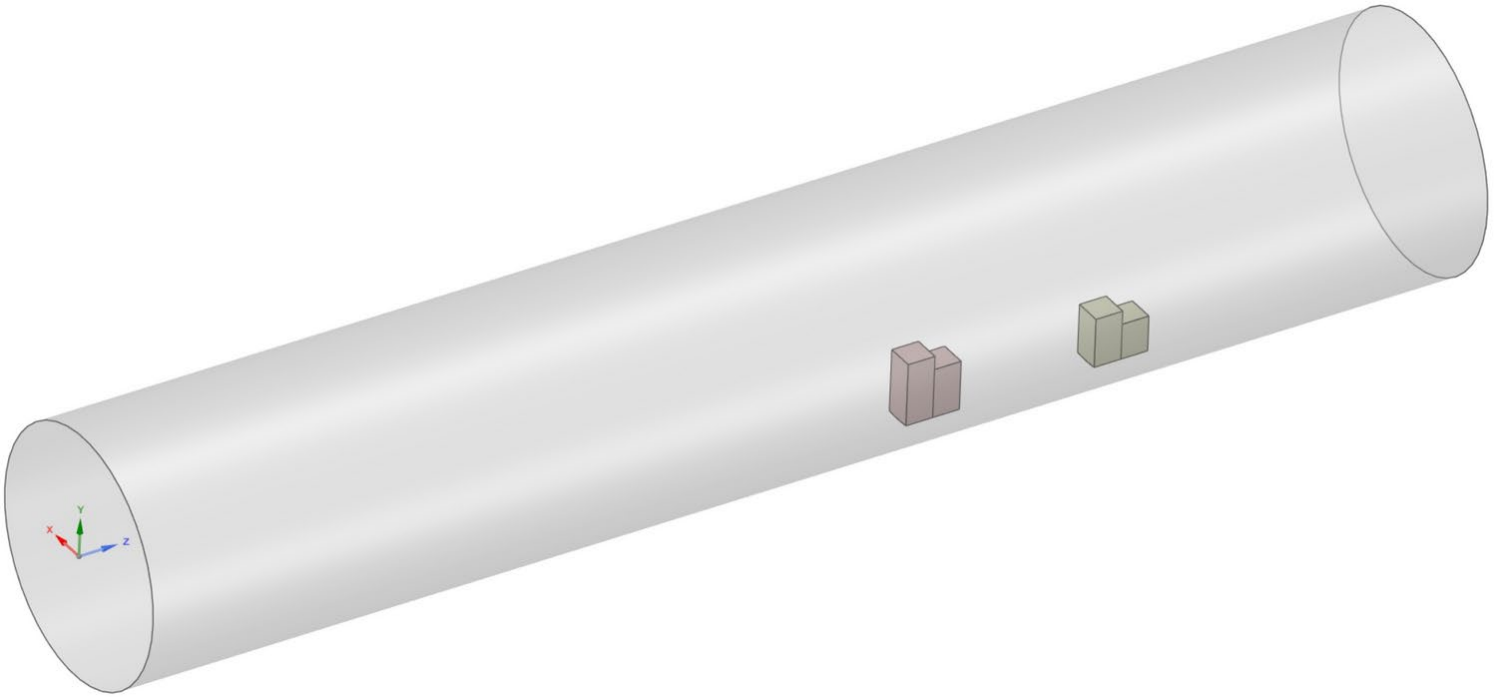
Schematic of the problem.

After placing a rectangular patch inside a circular pipe that conveys a fluid of density ($\rho$) as in Fig. 3, the drag coefficient ($C_d$) and inertial coefficient ($C_i$) come into play. $C_d$ characterizes the drag resistance, accounting for factors like the shape of the structure within the circular pipe, surface characteristics, and fluid dynamics. On the other hand, $C_i$ represents the inertial coefficient, capturing the added mass effect due to the interaction of the fluid with the submerged structure. The striking of the fluid in the inline direction along the *y*-axis can be described by the Morison’s equation, which provides an estimate of the inline force ($\varvec{F_i}$) acting on the submerged structure. Morison’s equation, when applied to this scenario, takes the form

25 $$\begin{aligned} \displaystyle \varvec{F_i}=0.5uC_dD_i|\varvec{u}| \rho +\rho \left( \frac{d\varvec{u}}{dt}\right) C_i\frac{\pi D_i^2}{4}. \end{aligned}$$

This equation is commonly used to analyze and design structures immersed in fluids, providing valuable insights into the impact of fluid forces on the structure. In response to an external force, piezoelectric materials exhibit the piezoelectric effect, leading to the generation of an electric charge and the consequent creation of a potential difference or voltage across the material. This phenomenon, known as the piezoelectric effect, underscores the material’s ability to convert mechanical stress into an electric charge. The voltage ($V_p$) elicited by a piezoelectric material in response to an applied force ($\varvec{F_i}$) can be quantified through the piezoelectric equation

26 $$\begin{aligned} \displaystyle V_p=\frac{|\varvec{F_i}|d_ct_pK_f}{\epsilon ^TA}. \end{aligned}$$

Here, $t_p$ represents the thickness of the piezoelectric material and $K_f$ represents the coupling factor. This equation concisely demonstrates the conversion of mechanical forces into electrical signals by piezoelectric materials. The power produced by the piezoelectric patch can be determined using the formula

27 $$\begin{aligned} \displaystyle P=V_p.I. \end{aligned}$$

Using Ohm’s law, the expression for the power produced in terms of $V_p$ is given as

28 $$\begin{aligned} \displaystyle P=\frac{V_p^2}{R_p}. \end{aligned}$$

The computation of harvested energy resulting from the interaction of fluid with a piezoelectric material can be determined using the formula

29 $$\begin{aligned} \displaystyle E=\frac{V_p^2}{R_p}t. \end{aligned}$$

### Boundary conditions and numerical setups

**Figure 4:**
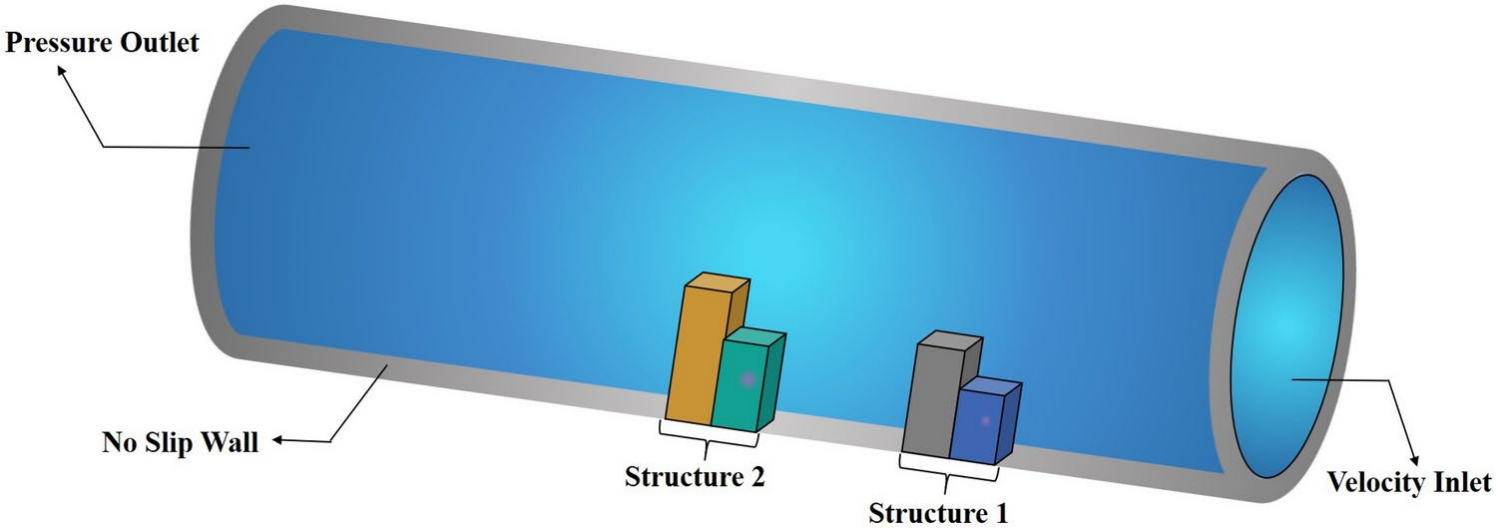
Boundary conditions used for the CFD simulations in ANSYS.

In this CFD model, the boundary conditions are carefully defined to ensure the accuracy of the simulation results. A velocity inlet is implemented at the inlet boundary, allowing the fluid to enter the pipe at a specified velocity. At the left boundary, a pressure outlet condition is established to maintain the desired pressure level as the fluid exits the system. Further, the pipe wall is treated with a wall boundary condition in conjunction with no-slip conditions, which ensures that the fluid velocity relative to the wall remains zero. This configuration effectively simulates the interaction between the fluid and the pipe wall, yielding reliable data on both the fluid’s behavior and the structural response (see Fig. 4).

The numerical simulations are conducted using ANSYS 2022 R1, employing both steady-state and transient pressure-based solvers to analyze the flow-induced vibrations. The steady-state solver is initially utilized to establish baseline flow characteristics before transitioning to transient analysis for a detailed examination of dynamic behaviors. To model the turbulent flow regime, the $k-\epsilon$ turbulence model is applied, complemented by structured mesh techniques that ensured a balance between computational efficiency and accuracy. Pressure-velocity coupling is managed through the PISO algorithm for transient simulations and the SIMPLE method for steady-state analysis. Spatial discretization schemes included the Least Squares Cell-Based method for gradients, PRESTO! for pressure interpolation, and the QUICK scheme for momentum equations, enhancing accuracy. A fixed time step of $\Delta t = 0.05$ seconds is adopted for transient simulations, with convergence criteria set at $1 \times 10^{-5}$ to ensure numerical stability. Initial and boundary conditions are meticulously defined to replicate the physical system under investigation, including inlet velocities and outlet pressures. This comprehensive numerical setup serves as a robust framework for analyzing the intricate fluid dynamics associated with the flow-induced vibrations, enabling insightful conclusions in engineering applications.

## ANSYS simulation process

**Figure 5:**
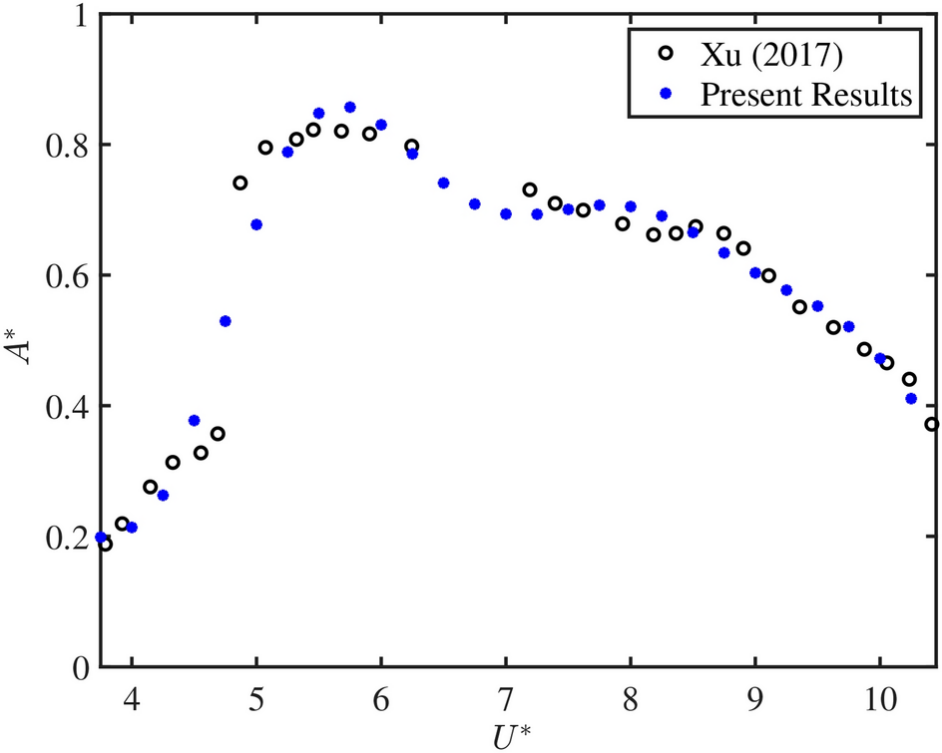
Comparing the present results with the results of Xu^25^.

Before going to present the results based on current simulations, it is necessary to validate our results with standard results available in the literature. For the same, Fig. 5 shows the comparison between the present results with the experimental results of Xu^25^. Here, the normalized amplitude variations $A^*$ associated with VIV response of the circular cylinder is plotted against the reduced velocity $U^*$. The cylinder considered here is having diameter 25 mm and submergence depth 820 mm. Remaining parameters values are taken same as mentioned in Xu^25^. The comparison reveals that the present ANSYS 2022 R1 based simulations matches well with the experimental results of Xu^25^.

**Figure 6:**
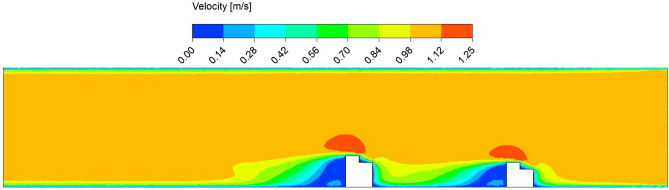
Contour velocity plot in the circular pipe conveying fluid, with a spacing distance of $d_2=0.202$ m.

**Figure 7:**
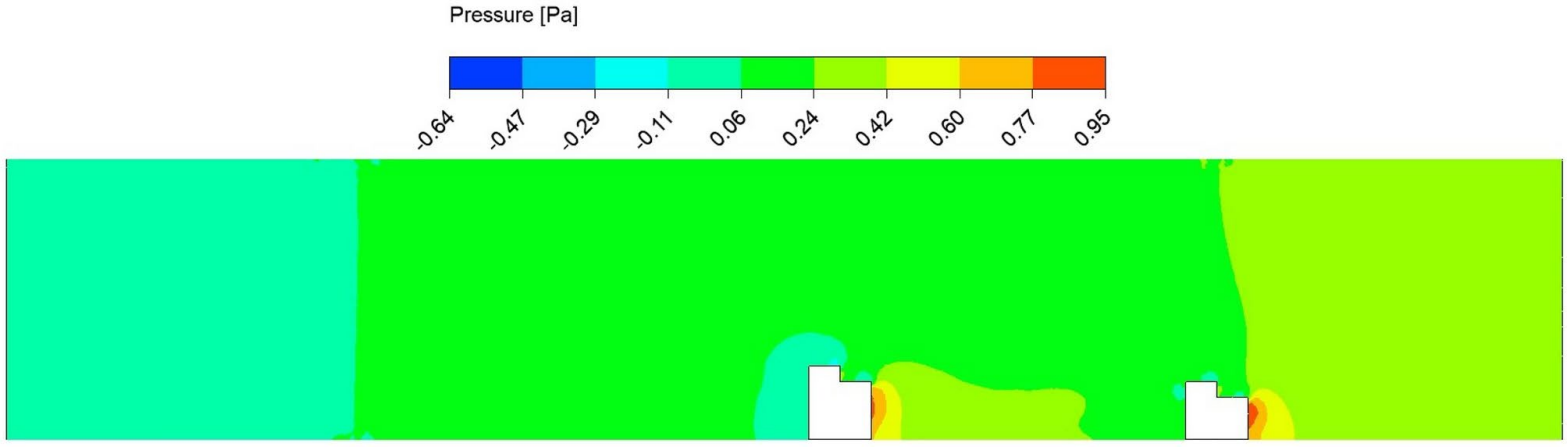
Contour pressure plot in the circular pipe conveying fluid, with a spacing distance of $d_2=0.202$ m.

**Figure 8:**
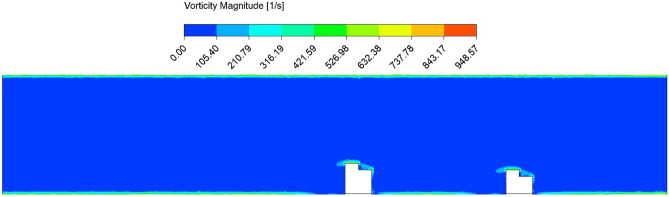
Vorticity magnitude contour plot in the circular pipe conveying fluid, with a spacing distance of $d_2=0.202$ m.

The fluid flow analysis precisely simulated the anticipated behavior of the fluids within the pipe. This enabled clear observation of the key phenomena, including the velocity and pressure of the fluid flow. In addition, analyzing the impact of structural spacing on PZT-5A output performance is crucial. We have considered three different distances between the structures $d_2 = 0.0505$ m, 0.101 m, and 0.202 m. The effect of each configuration on modal characteristics, structural deformation, and harmonic response is evaluated, providing insights into their influence on overall energy harvesting efficiency. The analysis aims to identify the optimal spacing for maximizing performance. Detailed results are presented for the case of $d_2 = 0.202$ m. In this study, the initial velocity of the fluid system is defined with 1.00 m/s along the *z*-axis, while all other directions remain inert. Simultaneously, the gauge pressure uniformly begins at 0 Pa across the computational domain. These carefully selected parameters lay the groundwork for the research investigation, facilitating a nuanced exploration of the fluid dynamics of the system. In Figs. 6 and 7, the velocity and pressure contour plots can be observed when the flowing fluid impacts the piezoelectric material at an average velocity of 1.00 m/s. These observations are conducted by selecting the *yz*-plane as the reference coordinate system. The velocity magnitude is high above the structures, and the value is lower at the back side of the beams. In addition, it is observed that the maximum velocity of the fluid flow is 1.25 m/s. The higher pressure region is the front wall of the structures, as the flow interacts directly and lowers the pressure in the back wall of the structures. The pressure contour has a maximum pressure value of 0.95 Pa near the front wall of the piezoelectric patches. Figure 8 presents the vorticity magnitude contour plot, which further illustrates the fluid dynamics around the structures. This plot reveals the intensity of vorticity, highlighting regions where vortices are forming due to flow separation and turbulence. The high vorticity areas correspond to the shedding of vortices from the structures, providing critical insights into the flow characteristics and their potential effects on energy harvesting performance. By analyzing the vorticity, a better understanding of how the flow interacts with the piezoelectric materials and the implications for optimizing their placement to enhance the energy harvesting efficiency.

**Figure 9:**
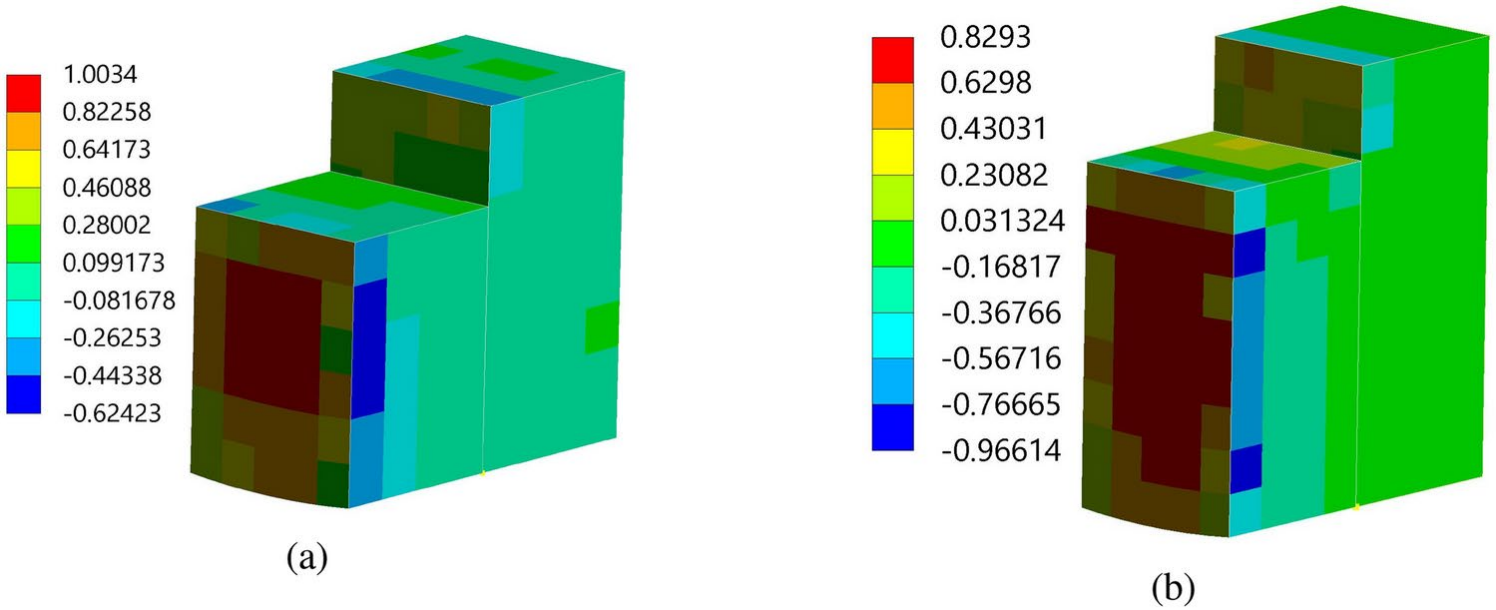
Imported pressure (Pa) plots on the piezoelectric patch and beam arrangement of (**a**) structure 1, and (**b**) structure 2, with a spacing distance of $d_2=0.202$ m.

### Static structural analysis

In this section, a static structural analysis is performed on beam structure arrangements equipped with PZT patches placed within a fluid-conveying pipe. The mechanical behavior is comprehended by examining deformation, stress distribution, and structural response to hydrodynamic loading. For this, fixed support constraints are applied on the bottom boundaries of both the beam structure arrangements. Then, the imported load is applied on all the faces of the beam structure with the piezoelectric patches.

**Figure 10:**
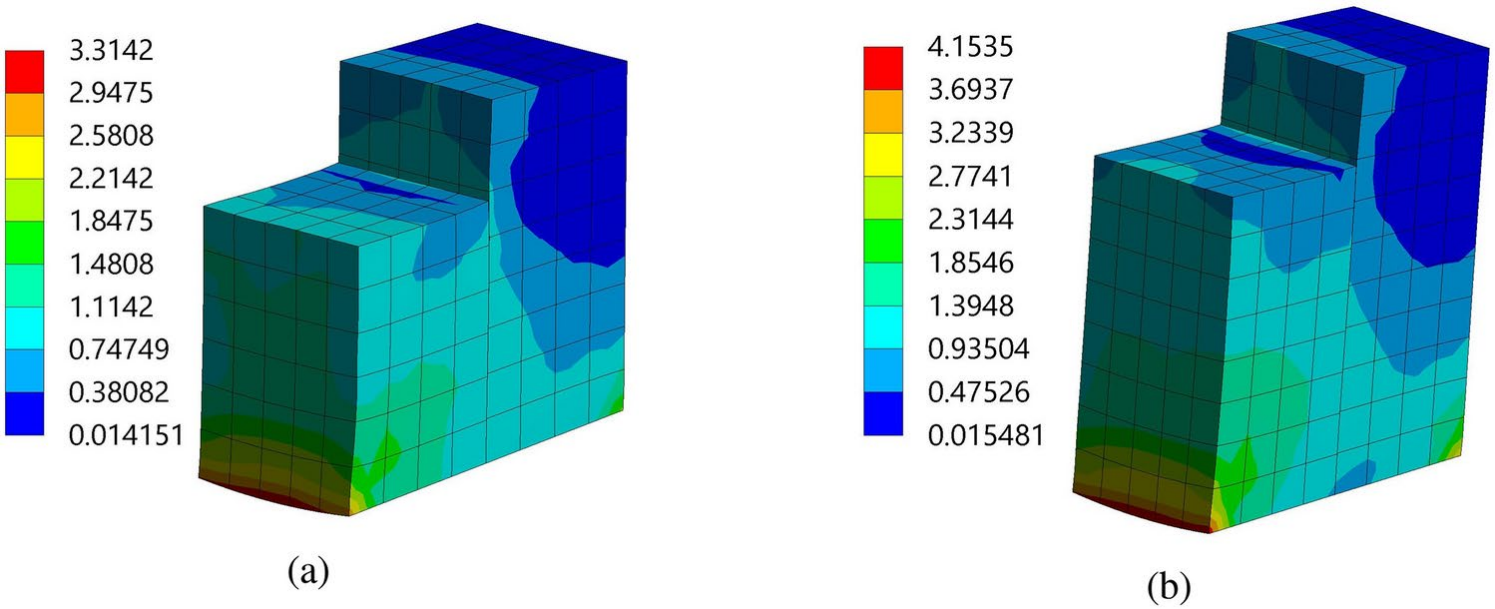
Equivalent (Von-Mises) Stress (Pa) plots on the piezoelectric patch and beam arrangement of (**a**) structure 1, and (**b**) structure 2, with a spacing distance of $d_2=0.202$ m.

**Figure 11:**
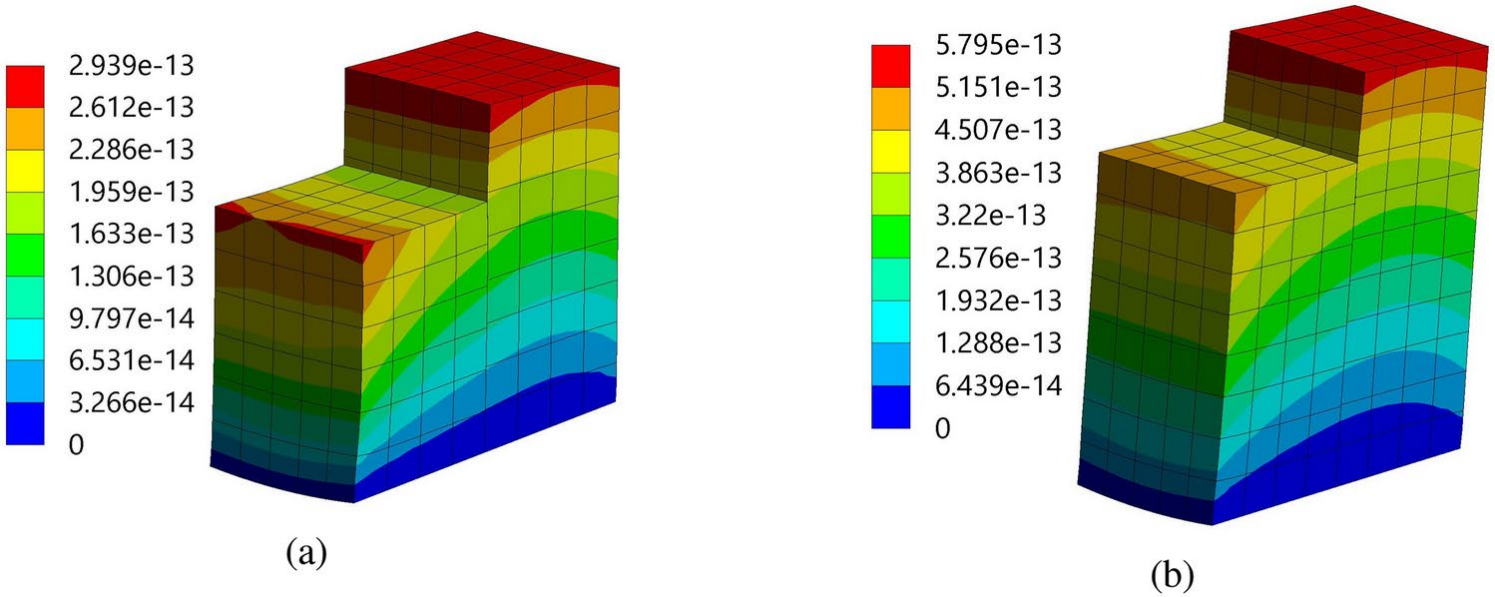
Total deformation (m) plots on the piezoelectric patch and beam arrangement of (**a**) structure 1, and (**b**) structure 2, with a spacing distance of $d_2=0.202$ m.

Figure 9 demonstrated the imported pressure on the piezoelectric patch and beam arrangement. It is observed that 1.003 Pa and 0.829 Pa are the maximum magnitudes imported on the structure 1 and 2, respectively. In addition, the magnitude of the pressure is high on the front wall of the PZT patches of both structures. Further, in the process of setting up the fluid-solid interface, a clear connection between them is established using the “Fluid-Solid Interface component” of ANSYS 2022 R1. This interface acts as the conduit for the information exchange between the fluid flow and structural analysis. For this, a fluid-solid interface is applied to all faces of the beam-PZT structures. Hereafter, to gain insight into the behavior of the beam-PZT structures under load, stress and deformation components are incorporated into the analysis. Figure 10 demonstrated the two structure’s Equivalent (Von-Mises) Stress plots. The Von-Misses stress values of the FE models varied from 0.0141 Pa to 3.3142 Pa for structure 1 and 0.0154Pa to 4.1535Pa for structure 2. Both models demonstrate a near similar high-stress concentration in the column’s front zone of about 3.3142 Pa to 4.1535 Pa. The high Von-Mises stresses are concentrated in the bottom section of the tee. Figure 11 illustrates the total deformation of the two structures 1 and 2, highlighting that the maximum amplitude of the total deformation is approximately $2.939\times 10^{-13}$ m and $5.795\times 10^{-13}$ m, respectively, indicating negligible changes in shape and excellent structural performance. The insights gained from this analysis will guide design optimization and improve performance for various applications, including flow sensing, structural health monitoring, and vibration control in fluid environments.

### Modal analysis

This study focuses on the modal analysis of two beam structures outfitted with PZT patches situated within a fluid-conveying pipe. Combining piezoelectric capabilities with fluid dynamics presents a nuanced challenge necessitating a comprehensive understanding of the dynamic responses of the structures. Placing these structures within a fluid environment introduces

**Figure 12:**
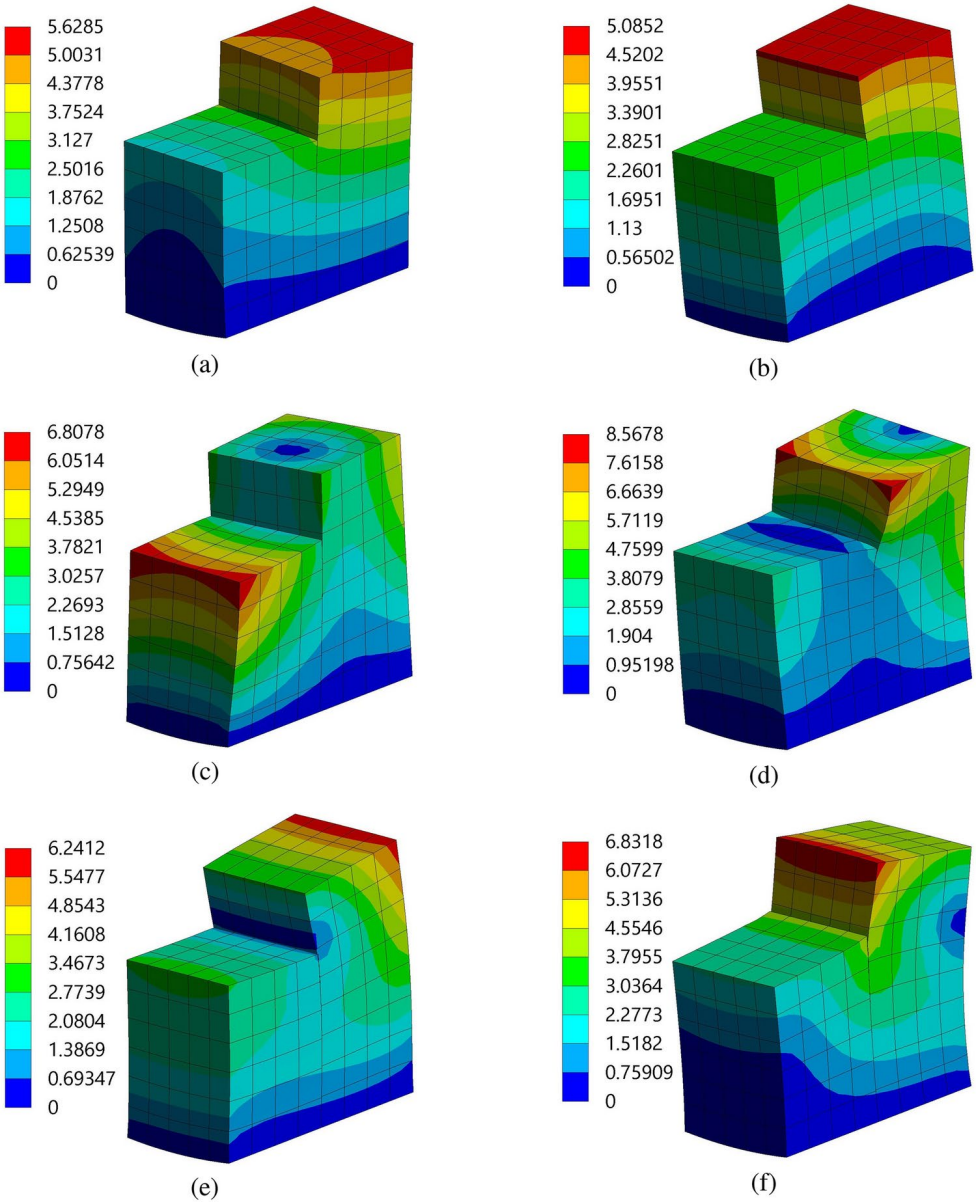
Mode shapes of the piezoelectric patch and beam arrangement of structure 1 at different frequencies (**a**) $f=12{,}107$ Hz, (**b**) $f=17{,}308$ Hz, (**c**) $f=21{,}438$ Hz, (**d**) $f=35{,}000$ Hz, (**e**) $f=36{,}275$ Hz, and (**f**) $f=39{,}694$ Hz, with a spacing distance of $d_2=0.202$ m. The unit in the colorbar is taken as meter.

additional complexities as fluid flow induces dynamic loading and impacts vibrational modes. This study aims to elucidate the natural frequencies, mode shapes, and other characteristics of these coupled systems through modal analysis. Such insights are critical to optimizing performance, ensuring structural integrity, and advancing engineering solutions in various applications.

**Figure 13:**
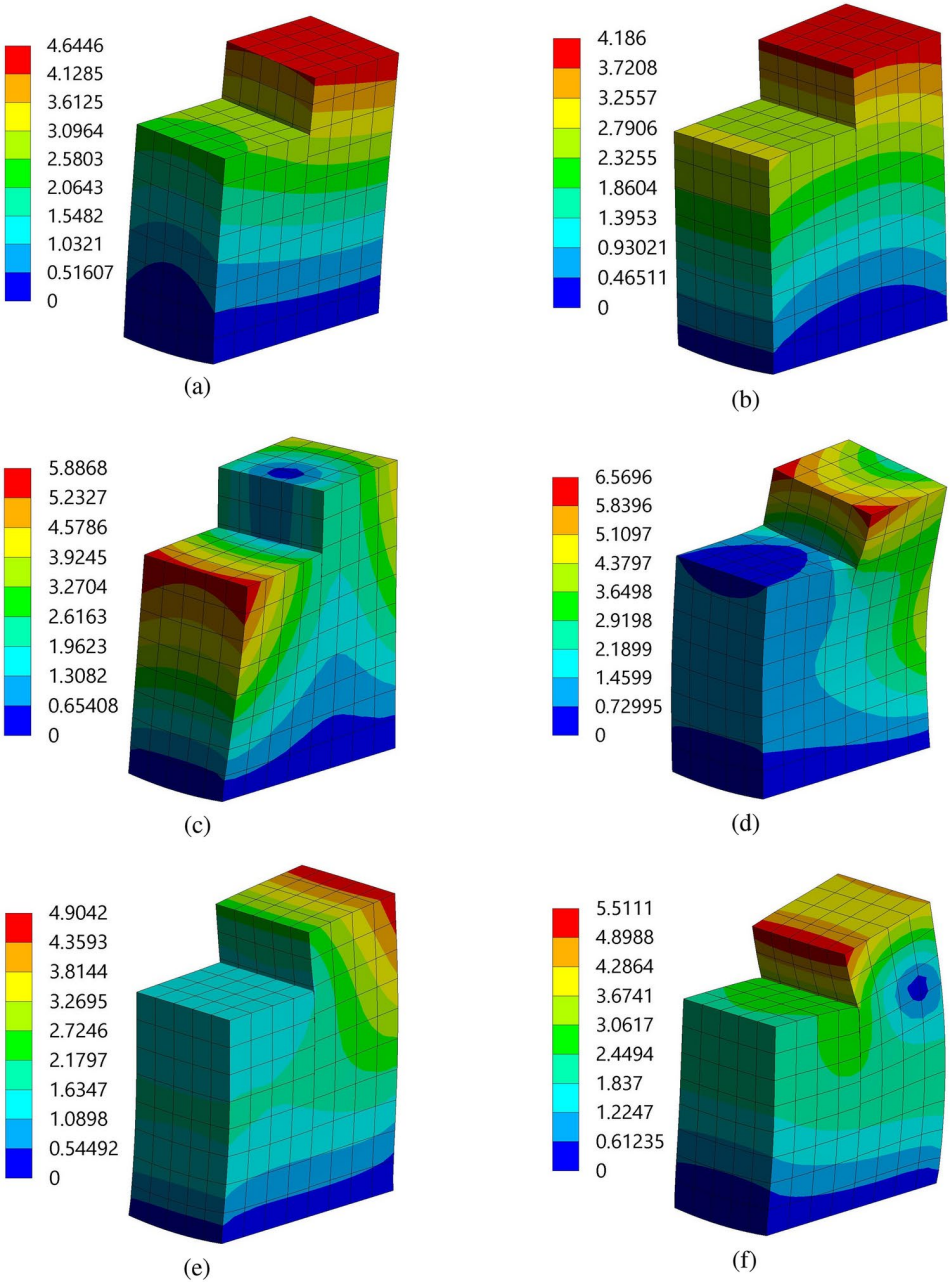
Mode shapes of the piezoelectric patch and beam arrangement of structure 2 at different frequencies (**a**) $f=7937.5$ Hz, (**b**) $f=12{,}142$ Hz, (**c**) $f=15{,}544$ Hz, (**d**) $f=28{,}112$ Hz, (**e**) $f=28{,}985$ Hz, and (**f**) $f=32{,}243$ Hz, with a spacing distance of $d_2=0.202$ m. The unit in the colorbar is taken as meter.

**Table 4:** Modal frequencies of structure 1 and structure 2 separated by distance $d_2=0.202$ m.

Mode	Frequency (Hz)	
	Structure 1	Structure 2
1	12, 107	7937.5
2	17, 308	12, 142
3	21, 438	15, 544
4	35, 000	28, 112
5	36, 275	28, 985
6	39, 694	32, 243

During modal analysis, it is imperative to specify the modes to be extracted, as each mode corresponds to a unique natural frequency and mode shape. These modes illustrate the diverse ways in which the structure can vibrate. Configuring the solver settings within ANSYS 2022 R1 ensures that the analysis runs efficiently and accurately. The natural frequencies corresponding to the modes 1 to 6 of the two structures are listed in Table 4. It is observed that the magnitude of natural frequencies are higher for structure 1 as compared to structure 2. These results indicate that structure 1 has greater stiffness and a better ability to resist dynamic loads, enhancing its energy capture performance. This stiffness plays a vital role in reducing the likelihood of deformation and potential damage due to vibrations. The meshing of the two structures is performed using ANSYS Meshing, with a hexahedral mesh type selected for its superior performance. Hexahedral elements have the feature of reduced integration, and Gauss point integration is used to enhance the precision of the analysis. As demonstrated in Figs. 12 and 13, using hexahedral elements with Gauss point integration significantly improves the accuracy of the first three modes. This improvement is also documented by Ahiwale et al.^26^. Further, it is crucial to understand the natural frequencies obtained because they indicate the specific frequencies at which the beams may resonate. Furthermore, the mode shapes obtained offer visual and quantitative insight into the vibration patterns at the aforementioned frequencies. These insights are essential to optimize the design, preventing resonance issues, and ensuring the reliability and efficiency of the system.

### Grid convergence analysis

Grid convergence analysis plays a crucial role in quantifying the numerical accuracy of the computational models. In the grid convergence study, the key parameters analyzed are natural frequency and voltage. By systematically refining the computational mesh, the numerical solution is expected to asymptotically approach to the exact solution of the governing partial differential equations, thereby minimizing discretization errors. In this study, the grid convergence is assessed following the guidelines prescribed in the ASME V & V $20-2009$ standards^27,28^. GCI analysis is performed using three grid levels $W^{(g)}_1$, $W^{(g)}_2$, and $W^{(g)}_3$ with a refinement ratio of 1.5, to confirm that the numerical solutions for natural frequency and voltage exhibit mesh independence. It estimates the discretization uncertainty in numerical simulations, calculated as follows:

1. **Set Up and Compare Three Grids with Different Resolutions**
  Run simulations on three grids with different resolutions. The grid refinement ratio *r* measures how much finer one grid is than another:
  30 $$\begin{aligned} r_{21} = \frac{W^{(g)}_2}{W^{(g)}_1}, \quad r_{32} = \frac{W^{(g)}_3}{W^{(g)}_2}. \end{aligned}$$
  Here, $r_{21} = r_{32} = 1.5$. The apparent order *m* is calculated as
  31 $$\begin{aligned} m = \frac{1}{\ln (r_{21})} \left[ \ln \left| \frac{\epsilon _{32}}{\epsilon _{21}} \right| + \chi (m) \right] , \end{aligned}$$
  where $\epsilon _{21} = \psi _2 - \psi _1$ and $\epsilon _{32} = \psi _3 - \psi _2$, with $\psi _k$ represents the variable of interest at each grid level. The function $\chi (m)$ is defined as
  32 $$\begin{aligned} \chi (m) = \ln \left( \frac{r_{21}^m - s}{r_{32}^m - s} \right) , \quad s = \sin \left( \frac{\epsilon _{32}}{\epsilon _{21}} \right) , \end{aligned}$$
  where $\chi (m) = 0$ when *r* is constant.
2. **Estimate Relative Errors**
  The relative errors between results on each pair of grids are calculated as follows
  33 $$\begin{aligned} e^{a}_{21} = \left| \frac{\psi _1 - \psi _2}{\psi _1} \right| , \quad e^{a}_{32} = \left| \frac{\psi _2 - \psi _3}{\psi _2} \right| . \end{aligned}$$
3. **Compute the GCI for Both Coarser and Finer Grids**
  The GCI values for the coarser and finer grids are computed using the formulae
  34 $$\begin{aligned} GCI^{21}_{Coarse} = \frac{1.25 \, e^{a}_{21} \, r^{m}_{21}}{r^{m}_{21} - 1}, \quad GCI^{32}_{Coarse} = \frac{1.25 \, e^{a}_{32} \, r^{m}_{32}}{r^{m}_{32} - 1}, \end{aligned}$$
  and for the finer grid as
  35 $$\begin{aligned} GCI^{21}_{Fine} = \frac{1.25 \, e^{a}_{21}}{r^{m}_{21} - 1}, \quad GCI^{32}_{Fine} = \frac{1.25 \, e^{a}_{32}}{r^{m}_{32} - 1}. \end{aligned}$$

These steps provide an estimate of the discretization uncertainty in our simulation results. As shown in Table 5, the fine-grid GCI values ($GCI_{Fine}$) range from 0.0000061 to 0.0101638, indicating that discretization errors are significantly reduced with mesh refinement. Similarly, the coarse-grid GCI values ($GCI_{Coarse}$) remain within acceptable bounds, further validating the consistency and accuracy of the numerical results across the tested mesh levels. Following this analysis, grid level $W_2^{(g)}$ is selected for subsequent simulations, as it provides an optimal balance between computational efficiency and accuracy. Thus, the results confirm that the numerical solution is sufficiently refined and that further mesh refinements will not yield significant changes, establishing the independence of the solution from mesh size.

**Table 5:** Discretization errors of the numerical solutions.

	Natural Frequency (Hz)		Voltage (V)	
	Structure 1	Structure 2	Structure 1	Structure 2
$\psi _1$	12079	8005	0.2071750	0.2054780
$\psi _2$	12107	7937.5	0.2072000	0.2054700
$\psi _3$	11908	7800	0.2065610	0.2053992
$\epsilon _{21}$	28.0	$-67.5$	0.0000250	$-0.0000080$
$\epsilon _{32}$	$-199.0$	$-137.5$	$-0.0006390$	$-0.0000708$
$r_{21}$	1.5	1.5	1.5	1.5
$r_{32}$	1.5	1.5	1.5	1.5
*m*	4.8366685	1.7547658	7.9933601	5.3775711
$\displaystyle e^{a}_{21}$	0.0023181	0.0084322	0.0001207	0.0000389
$\displaystyle e^{a}_{32}$	0.0164368	0.0173228	0.0030840	0.0003446
$\displaystyle GCI^{21}_{Coarse}$	0.0033721	0.0207041	0.0001570	0.0000549
$\displaystyle GCI^{21}_{Fine}$	0.0004745	0.0101638	0.0000061	0.0000062
$\displaystyle GCI_{Coarse}^{32}$	0.0239102	0.0425337	0.0040119	0.0004856
$\displaystyle GCI^{32}_{Fine}$	0.0033643	0.0208802	0.0001570	0.0000549

## Results and discussions

This section presents a detailed analysis of the hydrodynamics and performance of piezoelectric PZT-5A patches in turbulent fluid flow conditions. Two upright steel structures are installed inside a circular pipe through which fluids are conveyed. The interaction between the flowing fluids and the mounted structures induced vibrations in the PZT patches attached to the structures. These vibrations converted mechanical energy into electrical signals, providing insights into the functionality of fluid-based energy harvesters. The study aimed to identify the magnitudes and locations of the most significant stresses, thereby enhancing the understanding and optimization of the energy harvesting process. This emphasizes the development of a simulation model for the extraction of electrical energy using the piezoelectric material. Based on the feasibility study, two rectangular steel beam configurations with a PZT-5A layer embedded at the front are selected for analysis. Static structural analysis along with modal and harmonic response analysis are conducted using ANSYS Multiphysics 2022 R1 or ANSYS Mechanical 2022 R1 (https://www.ansys.com/) to evaluate the impact of flow-induced vibrations on the embedded PZT patches. In the aforementioned analysis, pressure, velocity, maximum deformation, and equivalent Mises stress are demonstrated in Sect. 3, and phase portrait, frequency, and voltage responses are described in Sect. 4. The computational resources used for this simulation included an Intel(R) Xeon(R) Silver 4116 CPU, running at a clock speed of 2.10 GHz, supported by 32 GB of RAM, and operating on a 64-bit system.

**Figure 14:**
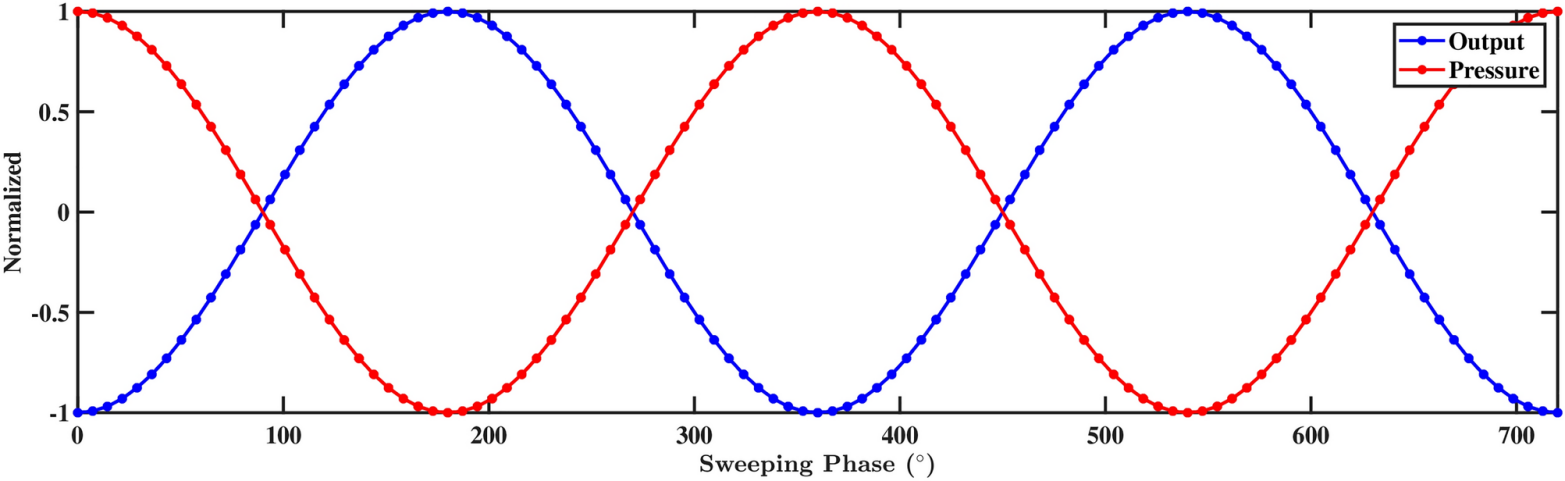
The phase shift between the pressure and output of the piezoelectric patch and beam arrangement.

**Figure 15:**
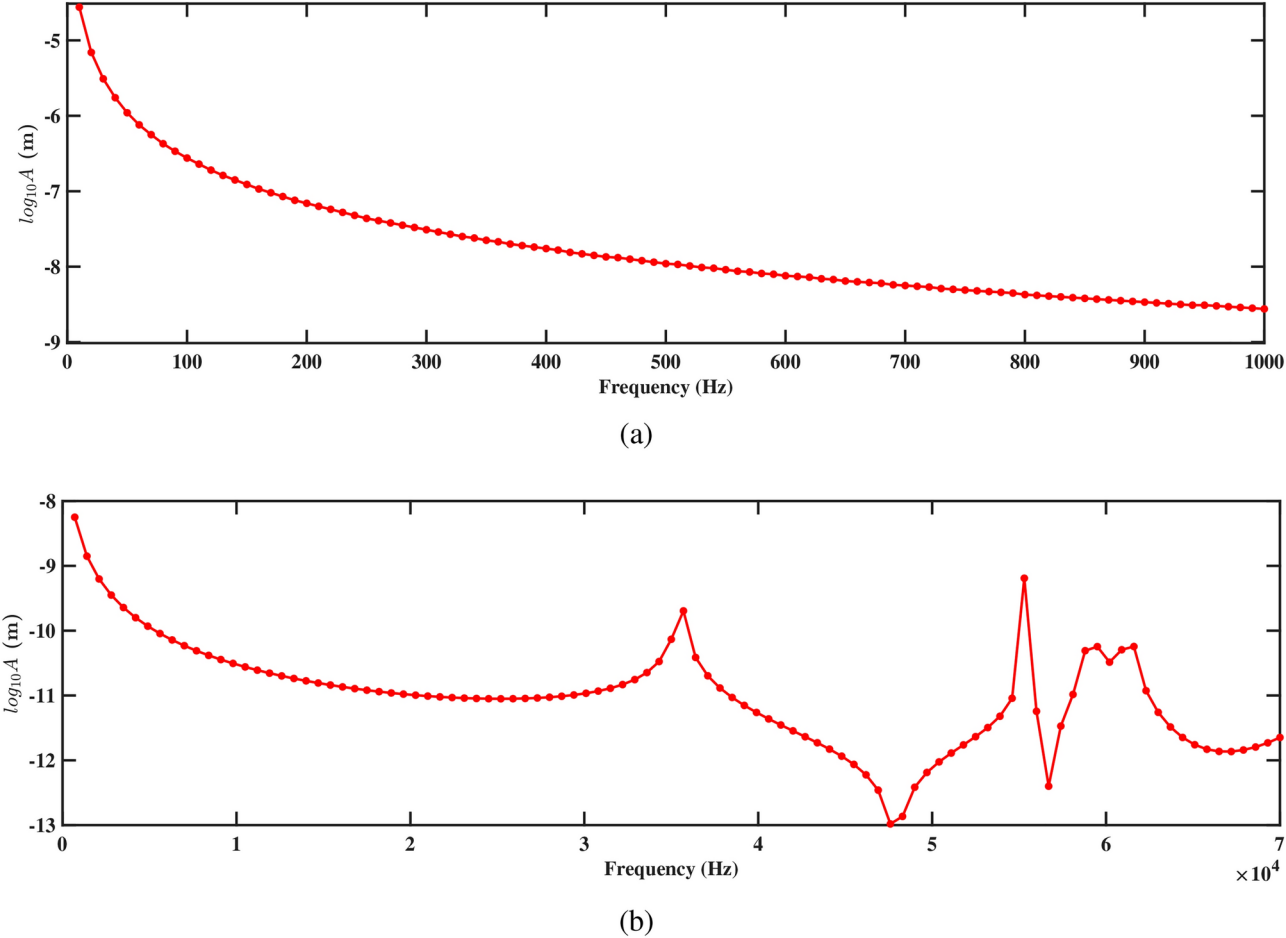
Amplitude with respect to the frequency corresponding to structure 1 (**a**) up to 1000 Hz, and (**b**) up to 70, 000 Hz, with a spacing distance of $d_2=0.202$ m.

When fluid flows through the pipe, it impinges on the PZT beams at an average speed of 1.00 m/s, creating pressure fluctuations and vortices. These interactions induce vibrations in the beams as a result of the fluid forces acting on their surfaces. The vibrating beams experience mechanical deformation, generating strain within the piezoelectric material, as shown in Sect. 3. To deepen understanding of the system’s dynamics, a harmonic response analysis is conducted by applying pressure directly to the front face of the PZT patches. This approach enables the evaluation of the responses of both structures to sinusoidal inputs across varying frequencies, along with the corresponding voltage outputs. The phase shift of pressure-output and frequency response analyses are performed at the tips of the beams, while the voltage response is measured at the tips of the PZT patches. Figure 14 represents the normalized pressure output plot, which illustrates the response of the deformed structure to the sweeping phase angle. The normalization is performed using max-absolute normalization, ensuring that the pressure values lie within the range of -1 to 1 for consistent comparison. This analysis captures the structure’s pressure variation over a full cycle of oscillation, highlighting both the positive and negative peaks in pressure output, which are indicative of the deformation’s impact on the system. The aforementioned analysis is performed at a frequency of 300 Hz, covering a duration of $720^\circ$. It is observed that as the pressure exerted value increases along the sweeping phase, there is a corresponding decrease in the output response. Conversely, when the pressure exerted value decreases, the output response increases. This observation signifies the sensitivity of the output response to variations in the pressure exerted along the sweeping phase. It emphasizes that changes in the applied pressure have a direct impact on the response of the system, highlighting the importance of regulating and optimizing the pressure exerted to achieve the desired outcomes in the analysis.

**Figure 16:**
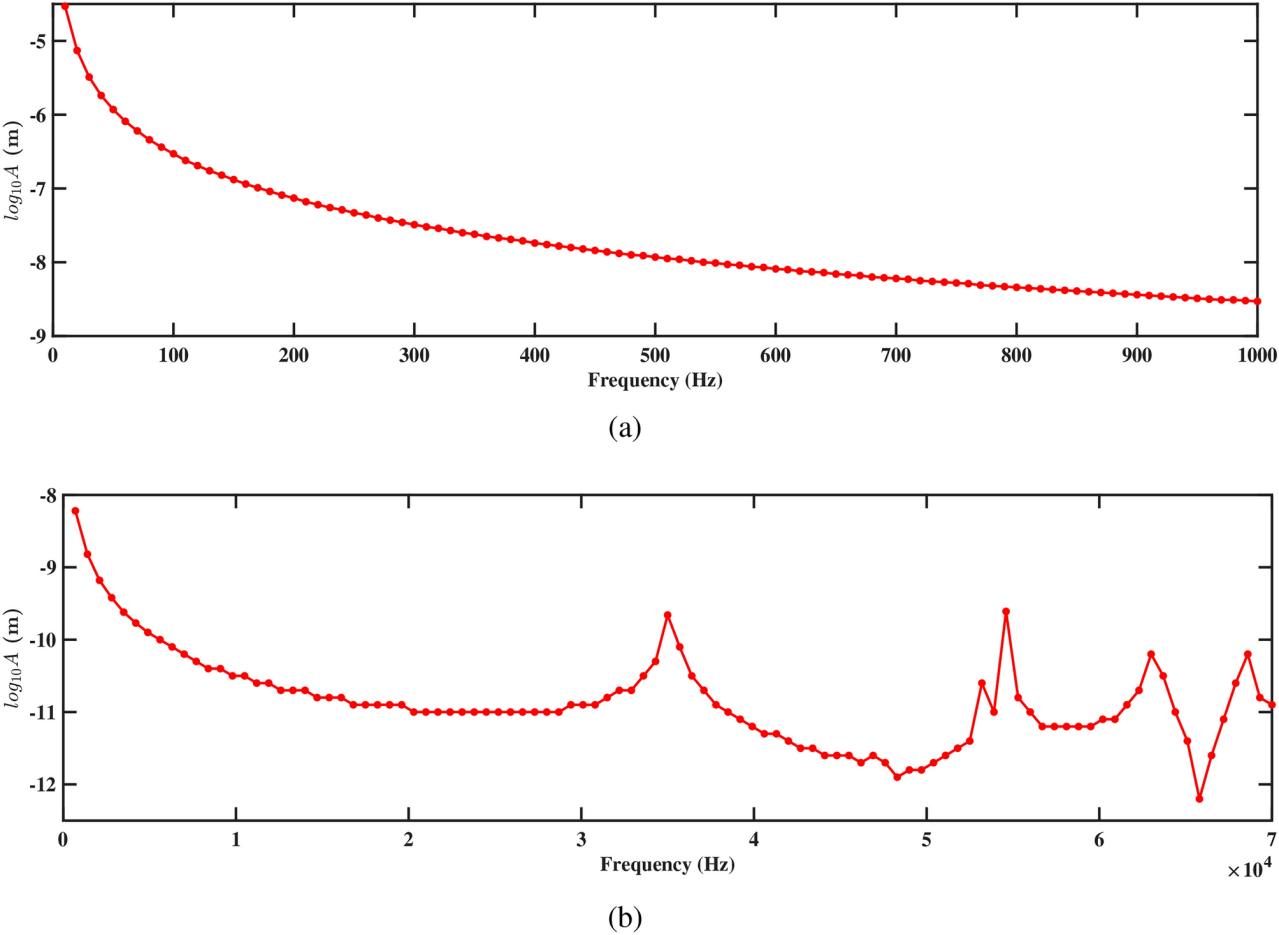
Amplitude with respect to the frequency corresponding to structure 2 (**a**) up to 1000 Hz, and (**b**) up to 70, 000 Hz, with a spacing distance of $d_2=0.202$ m.

Figures. 15 and 16 present the frequency response curves that plot the logarithm of amplitude against frequency for structures 1 and 2. The figures are divided into two parts: part (a) covers the frequency range from 0 Hz to 1000 Hz, while part (b) extends from 0 Hz to 70, 000 Hz. In the frequency range from 0 Hz to 1000 Hz, shown in part (a), the amplitude of the response gradually decreases. This suggests an inverse relationship with the direction of deformation, which indicates that as the frequency increases within the aforementioned range, the amplitude of the deformation decreases. This is important for understanding the structure’s response to low-frequency excitations and assessing its stability under various conditions. In the higher frequency range up to 70, 000 Hz shown in part (b), the response characteristics can vary more significantly, potentially highlighting multiple resonant peaks where the system’s natural frequencies align with the excitation frequencies. Further, it is observed that absence of peaks at the natural frequency in Figs. 15b and 16b arises because the natural frequencies are derived for the entire structure, while the frequency response is specifically measured at the tip of the beam. The natural frequency at the tip can differ from that of the overall structure due to localized effects and the unique dynamic behavior at this location. Understanding these responses across both low- and high-frequency ranges is crucial for optimizing the design and ensuring the structural integrity of the systems under study.

**Figure 17:**
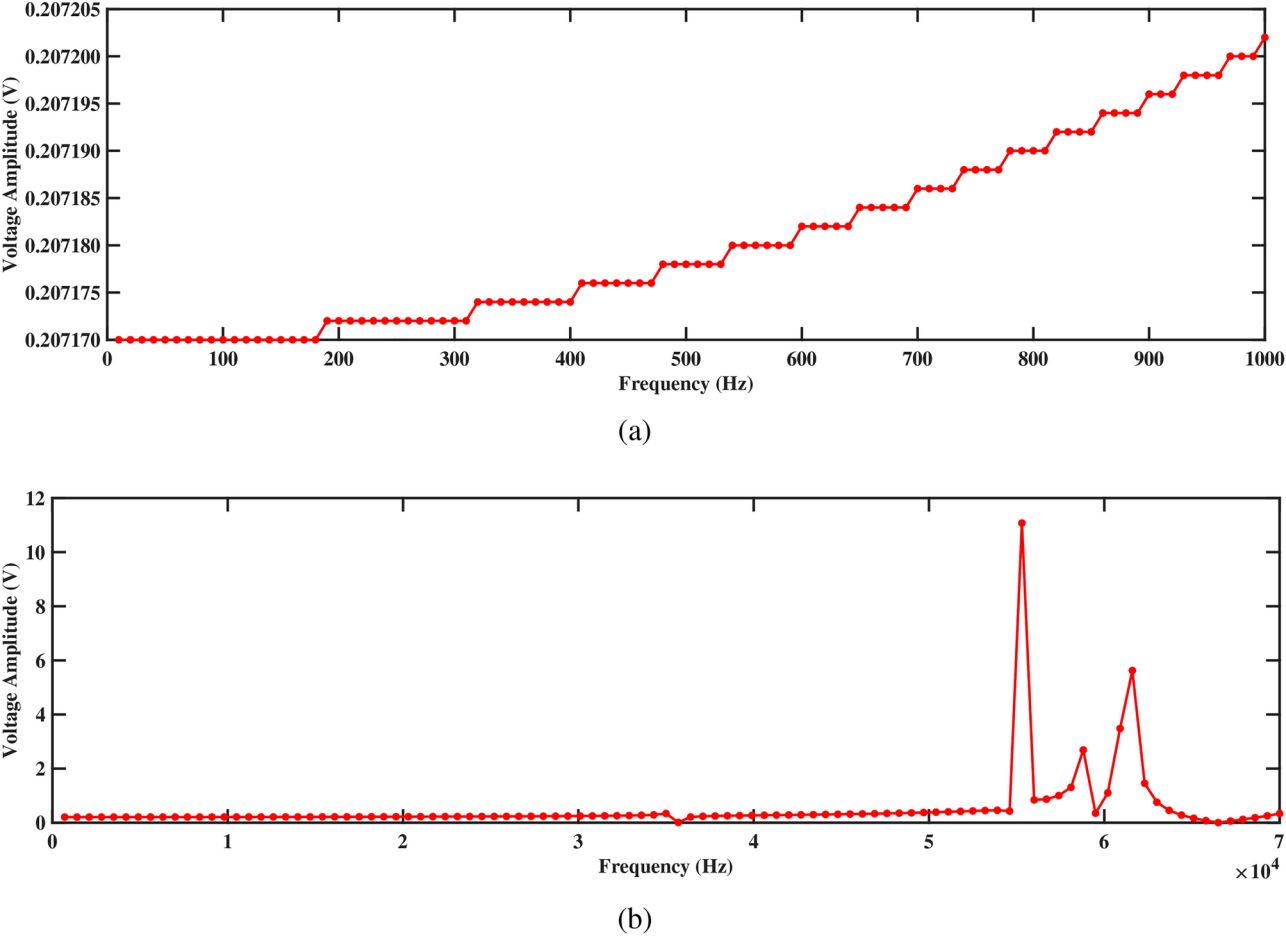
Voltage amplitude with respect to the frequency corresponding to structure 1 (**a**) up to 1000 Hz, and (**b**) up to 70, 000 Hz, with a spacing distance of $d_2=0.202$ m.

**Figure 18:**
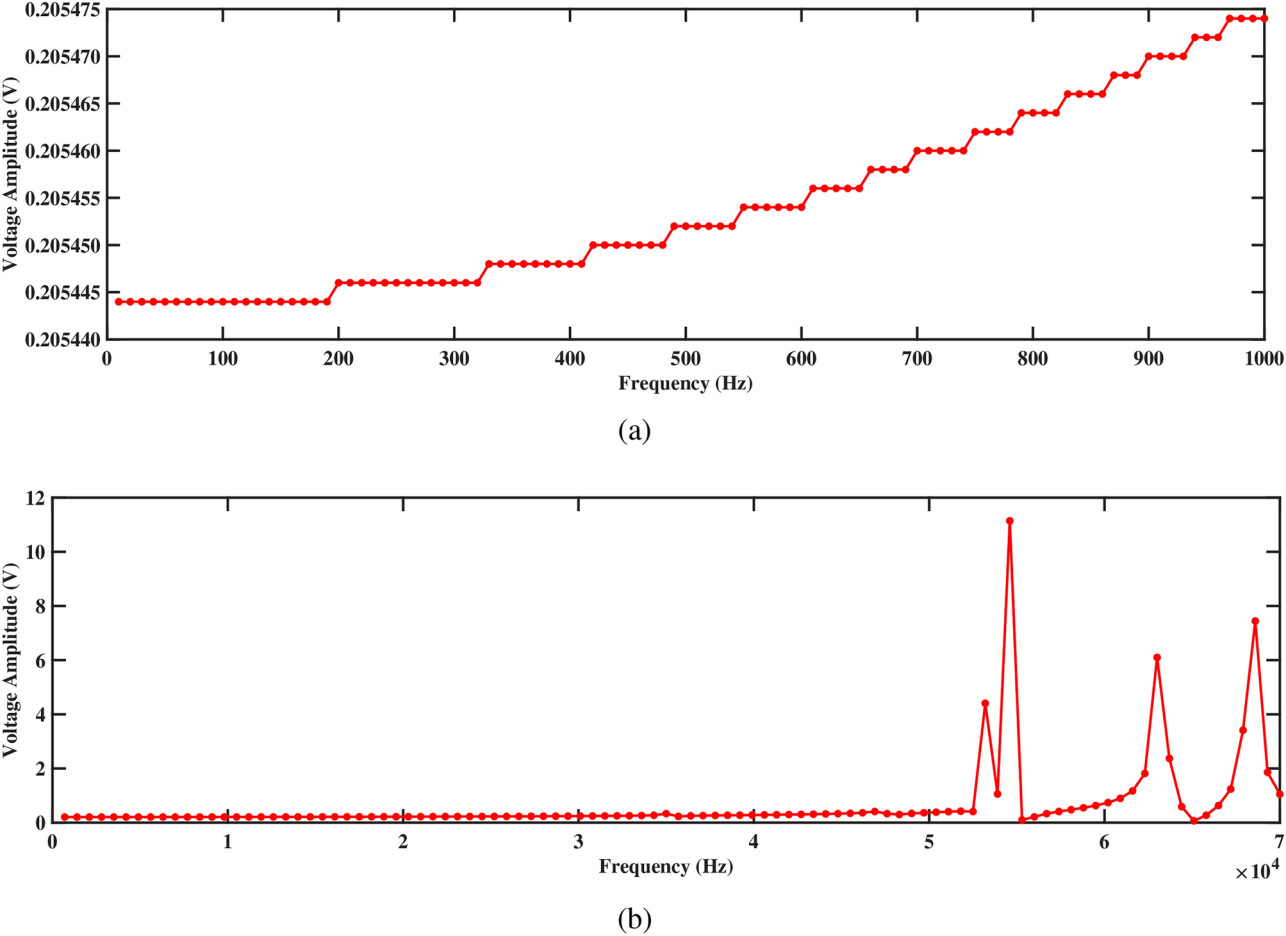
Voltage amplitude with respect to the frequency corresponding to structure 2 (**a**) up to 1000 Hz, and (**b**) up to 70, 000 Hz, with a spacing distance of $d_2=0.202$ m.

Figures 17 and 18 depict the voltage response characteristics of a piezoelectric system comprising two rectangular beams with attached PZT patches mounted within a circular pipe conveying fluid. In part (a), spanning frequencies from 0 Hz to 1000 Hz, the voltage amplitude exhibits an upward trend with increasing frequency, reaching maximum and minimum values of 0.20720 V and 0.20717 V, respectively, for beam 1 and 0.20547 V and 0.20544 V, respectively, for beam 2. This behavior underscores efficient energy conversion and delineates critical resonance peaks essential for comprehending the system’s dynamics under low-frequency excitations. The total voltage produced in this low-frequency range is 0.41264 V. Subsequently, part (b) extends the analysis to frequencies from 0 Hz to 70, 000 Hz, revealing multiple resonance peaks characterized by alternating peaks and troughs indicative of higher-order vibrational modes. Within this range, maximum and minimum voltages of 11.5 V and 0.2 V are observed for beam 1, and 11.5 V and 0.2 V are observed for beam 2, respectively. In addition, the total voltage produced in this high frequency range is 23.0 V, demonstrating their combined electrical output potential. Furthermore, the plots facilitate optimization endeavors by identifying optimal frequency ranges for energy conversion, enabling strategic placement and configuration of PZT patches, and safeguarding against detrimental resonance frequencies, thus enhancing overall performance and reliability.

The investigation into voltage output from the two-beam piezoelectric system, alongside previous research conducted by Yadav et al.^29^, sheds light on the advancements made in energy harvesting efficiency. Through this analysis, the maximum voltage value of 0.41264 V is observed from the structures across a broad frequency range. This significant enhancement in voltage output, compared to previous single-beam configurations, underscores the efficacy of the aforementioned approach in optimizing energy harvesting from fluid-induced vibrations. The strategic placement of PZT patches on multiple beams within the circular pipe, coupled with careful consideration of resonance phenomena, contributes to the observed increase in voltage generation. These findings highlight the potential of our two-beam system to offer superior performance in various applications that require enhanced energy harvesting capabilities.

### Impact of structural positioning on PZT-5A output performance

The positioning of structures plays a crucial role in determining the output performance of the PZT-5A piezoelectric material, as it directly influences both mechanical interactions and the resultant electrical responses. In this study, three distinct cases are examined with varying distances $d_2=0.0505$ m, 0.101 m, and 0.202 m between the structures. These configurations are chosen to explore how changes in separation distance affect the natural frequencies, frequency response and voltage outputs of the piezoelectric system.

#### Case I: Spacing distance $d_2 = 0.202$ m

At the largest separation distance of $d_2=0.202$ m, a significant increase in natural frequencies is observed for both structures. Structure 1’s first mode frequency rises to 12, 107 Hz, while structure 2’s increases to 7937.5 Hz. This increase is primarily due to the reduced mechanical interaction between the structures, which leads to higher stiffness and elevated natural frequencies. As the separation increases, the structures behave more independently, allowing for improved resonance characteristics. This trend is even more pronounced in higher modes (see details in Table 4), with structure 1 reaching 39, 694 Hz at the 6th mode and structure 2 achieving 32, 243 Hz. The larger spacing minimizes interference, enhancing their ability to resonate effectively and reducing energy dissipation caused by damping effects.

Figures 15a and 16a demonstrate the frequency-amplitude response of structure 1 and structure 2, respectively, up to 1000 Hz. Both structures exhibit a gradual decrease in amplitude as the frequency increases. The frequency response curve, particularly in the lower frequency range, indicates that the interaction between the two structures becomes weaker, allowing them to vibrate more freely and with less interference. Despite the lower amplitude response in the logarithmic scale, the voltage output at this frequency is significantly improved compared to previous cases, indicating enhanced energy harvesting efficiency at this larger spacing. Kang et al.^30^ reported the similar observations. At higher frequencies 70, 000 Hz, as shown in Figs. 15b and 16b, the resonance peaks are more pronounced. These peaks indicate that the structures can resonate more effectively due to the reduced mechanical coupling. The resonant peaks are sharper, suggesting less energy dissipation and higher energy transfer to the piezoelectric patches. The increased separation allows both structures to maintain effective resonance behavior, leading to improved strain and higher voltage output at these high frequencies.

Both structures also exhibit significantly improved voltage output at 1000 Hz, as shown in Figs. 17a and 18a, generating approximately 0.20720 V for structure 1 and 0.20547 V for structure 2. Figures 17b and 18b depict the voltage amplitude of structures 1 and 2 at frequencies of 70, 000 Hz. Both structures increase their output in this frequency range, reaching around 11 V. This higher voltage output results from efficient energy transfer to the piezoelectric patches, facilitated by the balanced configuration that maximizes strain without excessive damping. Overall, the larger separation enhances performance by allowing for more independent operation, resulting in better energy harvesting efficiency for both structures.

#### Case II: Spacing distance $d_2 = 0.101$ m

When the separation distance increases to $d_2=0.101$ m, the natural frequencies show a slight increase compared to the third case. For structure 1, the fundamental mode is 10350 Hz, while for structure 2, it is 6572.5 Hz, indicating a marginal rise. This suggests that the increased spacing reduces the coupling effects between the structures, allowing for more independent vibration. As a result, the stiffness slightly increases, which leads to this moderate rise in frequencies. The higher mode frequencies as provided in Table 6, remain close to those observed in the previous case, with structure 1 reaching 31, 460 Hz and structure 2 at 25, 610 Hz.

**Table 6:** Modal frequencies of structure 1 and structure 2 separated by distance $d_2=0.101$ m.

Mode	Frequency (Hz)	
	Structure 1	Structure 2
1	10, 350	6572.5
2	12, 909	8886.3
3	14, 556	10, 890
4	28, 276	23, 278
5	29, 291	23, 920
6	31, 460	25, 610

**Figure 19:**
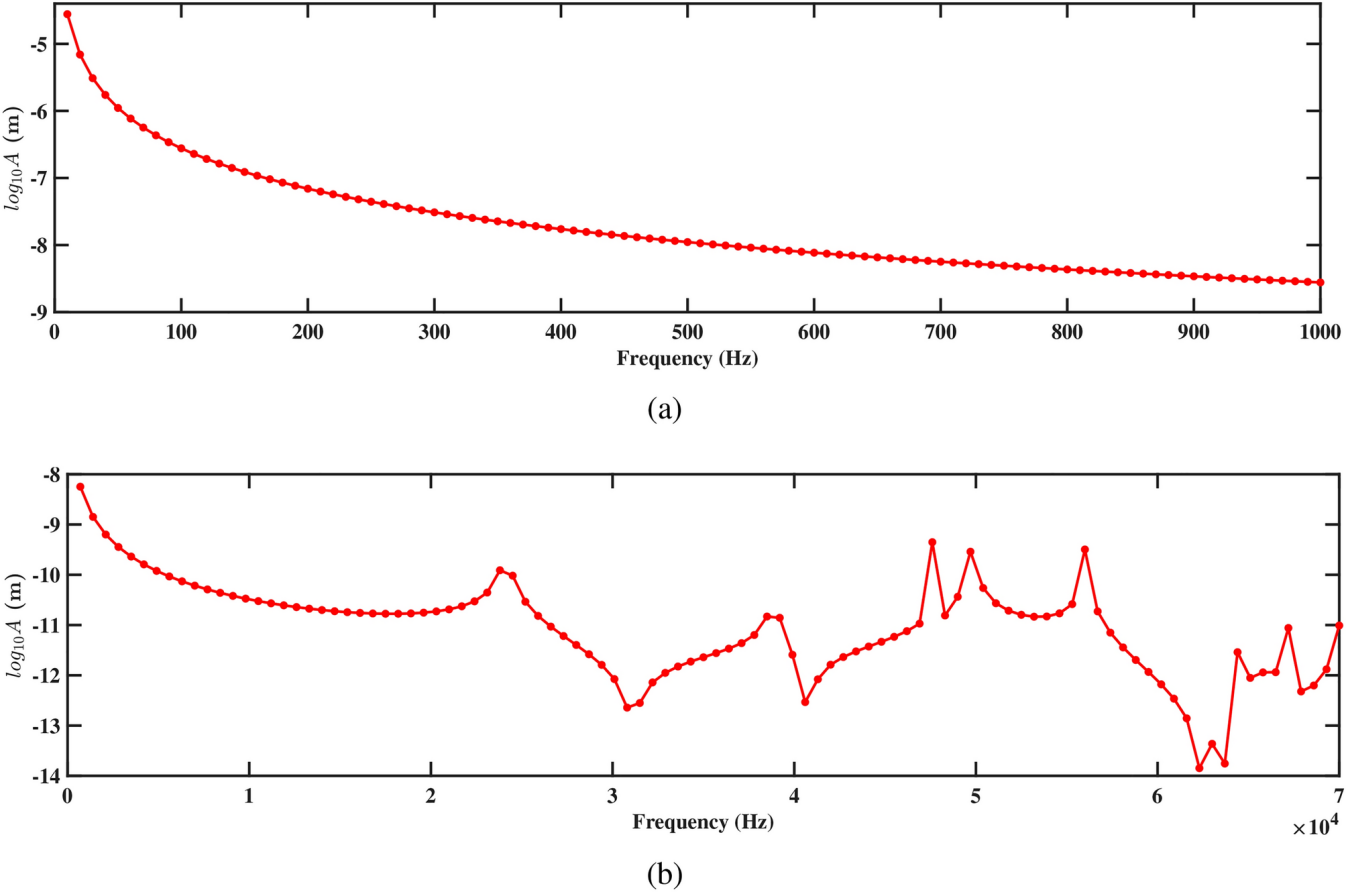
Amplitude with respect to the frequency corresponding to structure 1 (**a**) up to 1000 Hz, and (**b**) up to 70, 000 Hz, with a spacing distance of $d_2=0.101$ m.

**Figure 20:**
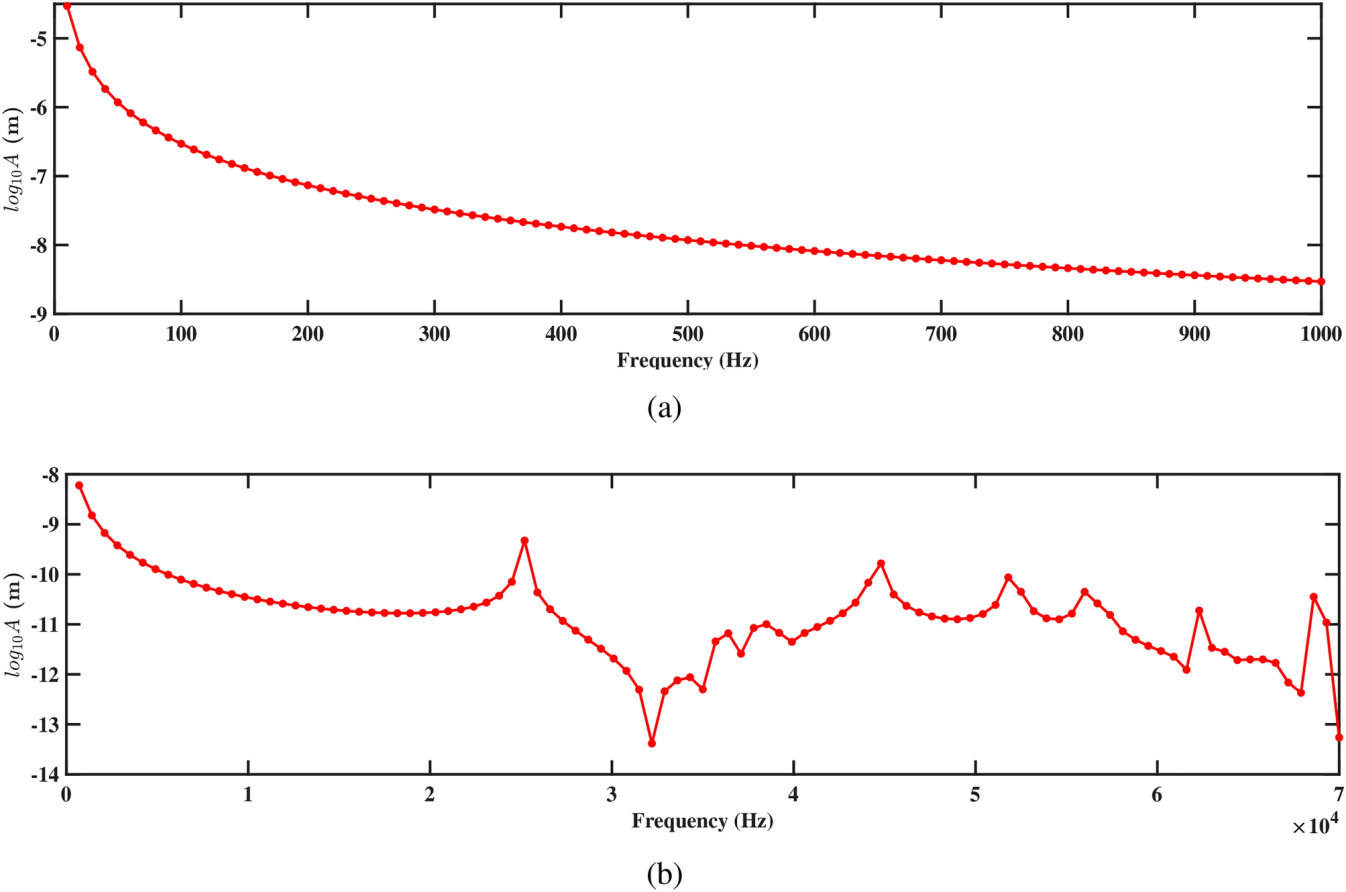
Amplitude with respect to the frequency corresponding to structure 2 (**a**) up to 1000 Hz, and (**b**) up to 70, 000 Hz, with a spacing distance of $d_2=0.101$ m.

The frequency-amplitude response reveals the influence of increased separation distance, particularly at 1000 Hz and 70, 000 Hz. Figures 19a and 20a show the amplitude response for structures 1 and 2 at frequencies up to 1000 Hz. In both figures, the amplitude decreases smoothly with the increasing frequency, showing a steady reduction in vibrational energy. It is observed that at lower frequencies, the structure experiences less vibration, and the surrounding fluids help in reducing the motion. The absence of sharp peaks indicates that the energy from the fluid flow is not strongly affecting the structure in this range. Figures 19b and 20b depict the amplitude response up to 70, 000 Hz for both the structures. It is observed that the smoothness of the frequency-amplitude response curve decreases with the emergence of several peaks. The vibration of the structure is more at these peak frequencies due to less effective damping, and absorb more energy from the fluid flow as the frequency increases. These results illustrated the flow-induction forces affect the vibrations of the piezoelectric energy harvester.

**Figure 21:**
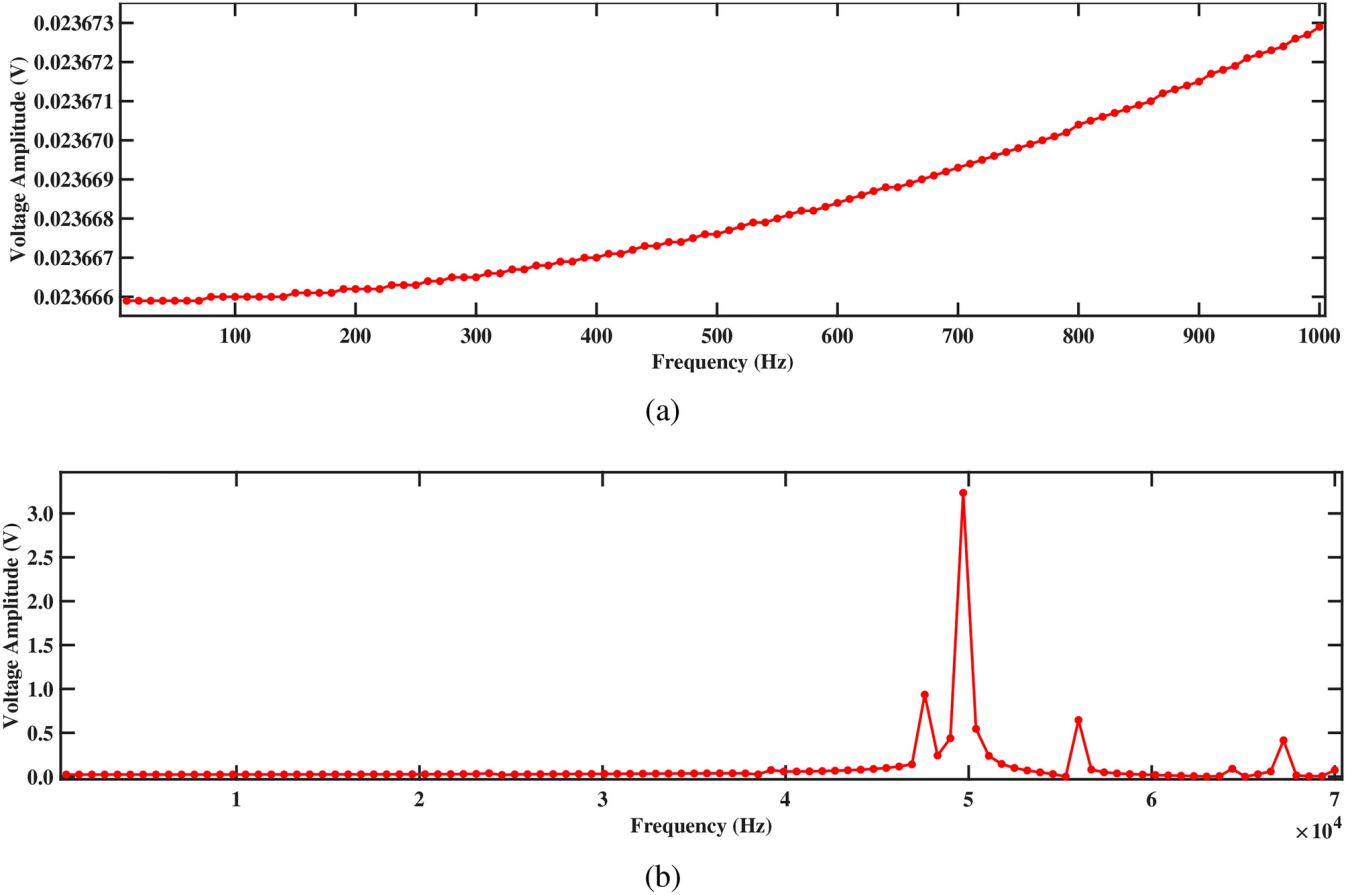
Voltage amplitude with respect to the frequency corresponding to structure 1 (**a**) up to 1000 Hz, and (**b**) up to 70, 000 Hz, with a spacing distance of $d_2=0.101$ m.

**Figure 22:**
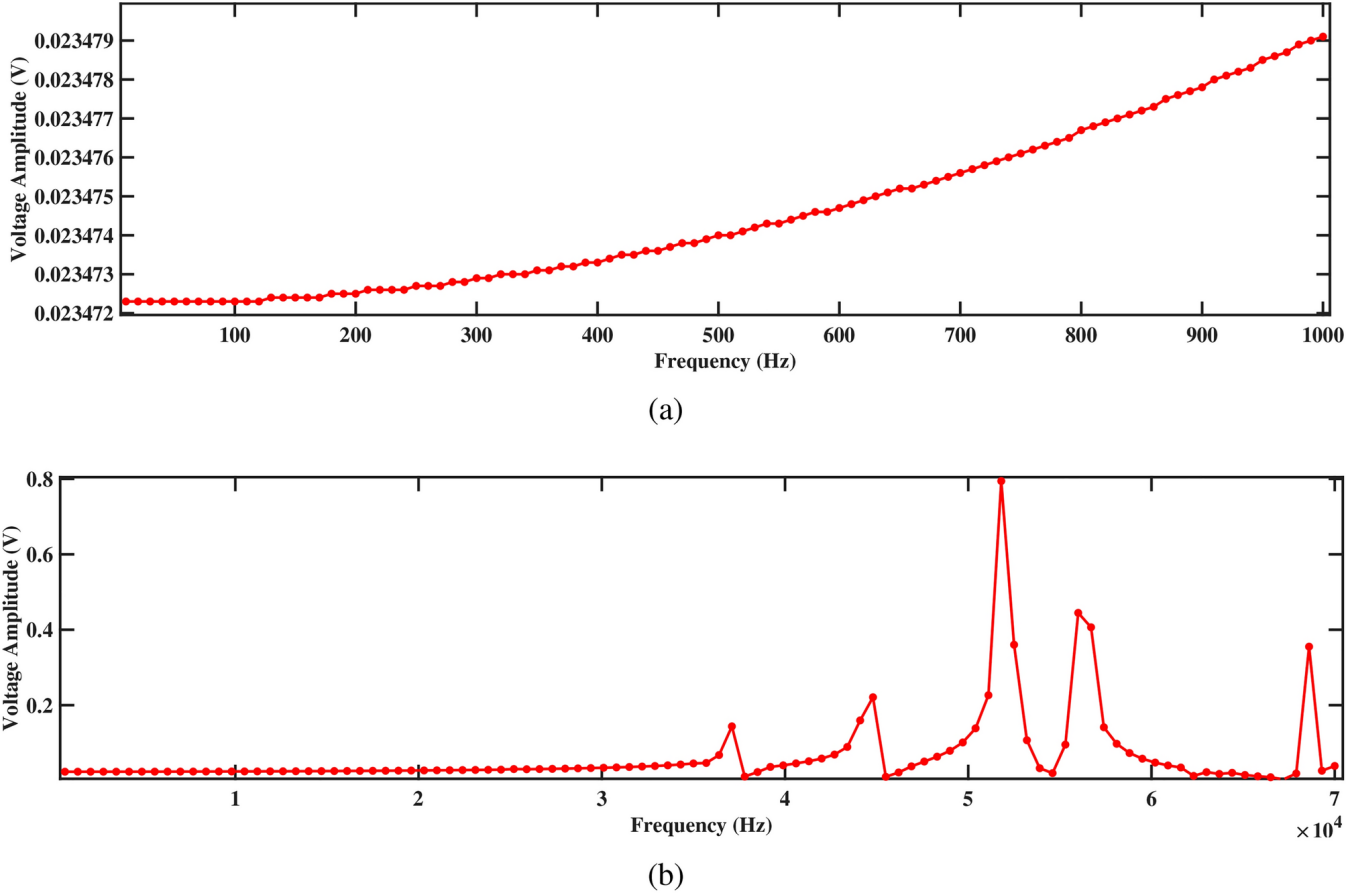
Voltage amplitude with respect to the frequency corresponding to structure 2 (**a**) up to 1000 Hz, and (**b**) up to 70, 000 Hz, with a spacing distance of $d_2=0.101$ m.

Further, the impact on voltage output is more pronounced, as depicted in Figs. 21 and 22. Figures 21a and 22a illustrate the voltage amplitude of structure 1 and structure 2 across the frequency range of 0 to 1000 Hz, providing a detailed comparison of their performance at lower frequencies. At 1000 Hz, both structures show a significant drop in voltage, with structure 1 generating 0.02367 V and structure 2 producing 0.02347 V. This reduction can be attributed to the weakened mechanical coupling, which limits the strain transfer to the piezoelectric patches. As the distance between the structures increases, their interaction weakens. This reduced interaction leads to a significant decline in energy transfer efficiency. Figures 21b and 22b demonstrate the voltage amplitude between structure 1 and structure 2 up to 70, 000 Hz. It is observed that structure 1 produces 3.23488 V, while structure 2 only generates 0.79430 V, highlighting a significant disparity in voltage output between the two structures. This large difference in voltage output at high frequency is likely due to the weakened resonance in structure 2, which struggles to maintain sufficient strain and energy transfer at this intermediate separation. Structure 1, with its slightly higher natural frequency and stiffness, retains better performance, while structure 2 suffers from reduced energy harvesting efficiency at this spacing.

**Table 7:** Modal frequencies of structure 1 and structure 2 separated by distance $d_2=0.0505$ m.

Mode	Frequency (Hz)	
	Structure 1	Structure 2
1	10, 359	6564.8
2	12, 921	8878.1
3	14, 572	10, 879
4	28, 321	23, 264
5	29, 347	23, 896
6	31, 486	25, 588

#### Case III: Spacing distance $d_2 = 0.0505$ m

At the closest spacing of $d_2=0.0505$ m, the natural frequencies of both structures are lower than those observed at larger separations. Structure 1 has a fundamental frequency of 10, 359 Hz, while structure 2’s frequency is 6564.8 Hz. This difference indicates a strong coupling effect between the structures; however, the close proximity leads to reduced overall stiffness and lower resonance frequencies. The natural frequencies for the higher modes are provided in Table 7. As the mode number increases, the frequencies rise, with structure 1 reaching 31, 486 Hz and structure 2 at 25588 Hz. The increased mechanical damping resulting from their proximity limits the resonance potential of both structures.

**Figure 23:**
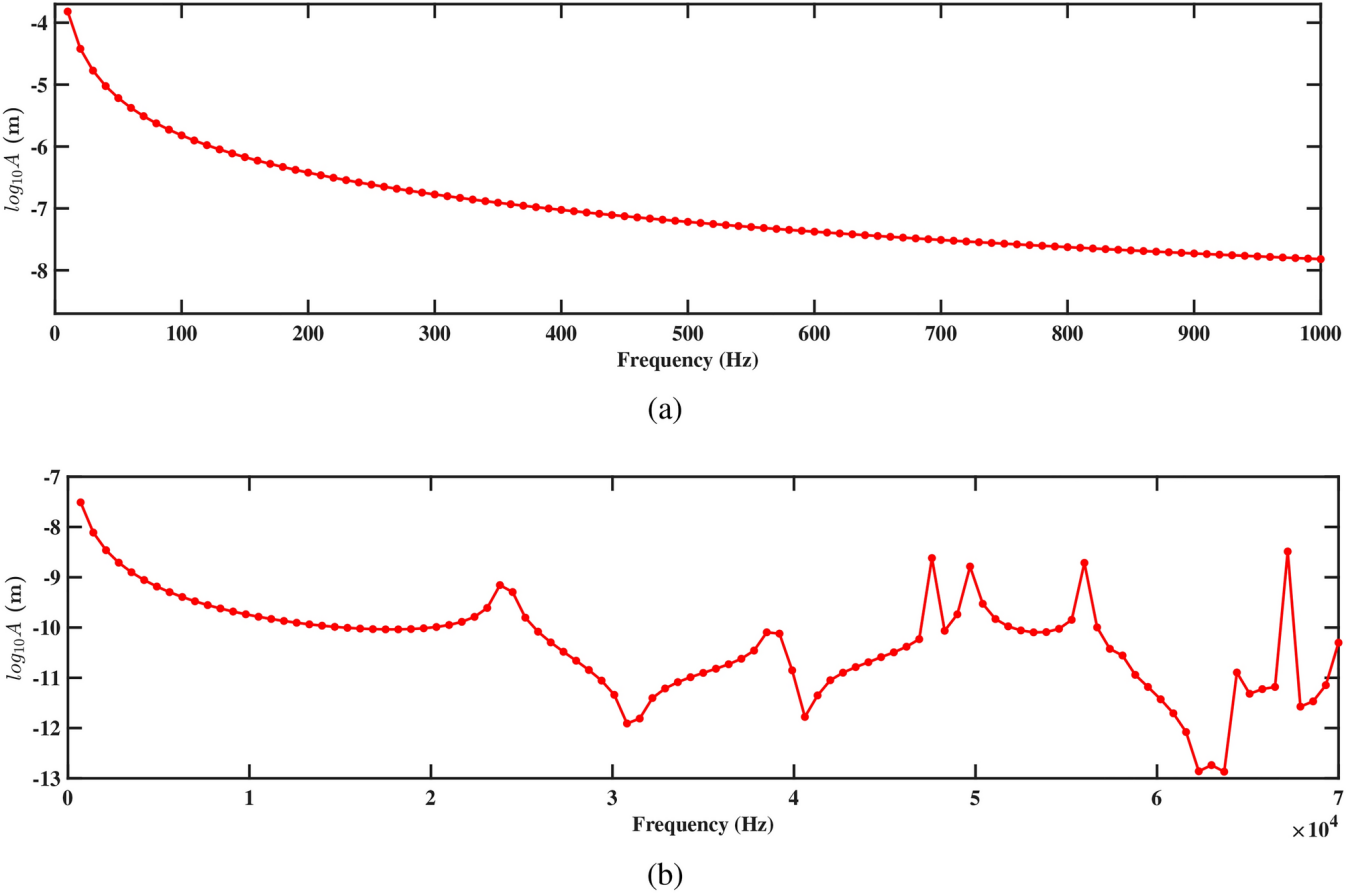
Amplitude with respect to the frequency corresponding to structure 1 (**a**) up to 1000 Hz, and (**b**) up to 70, 000 Hz, with a spacing distance of $d_2=0.0505$ m.

**Figure 24:**
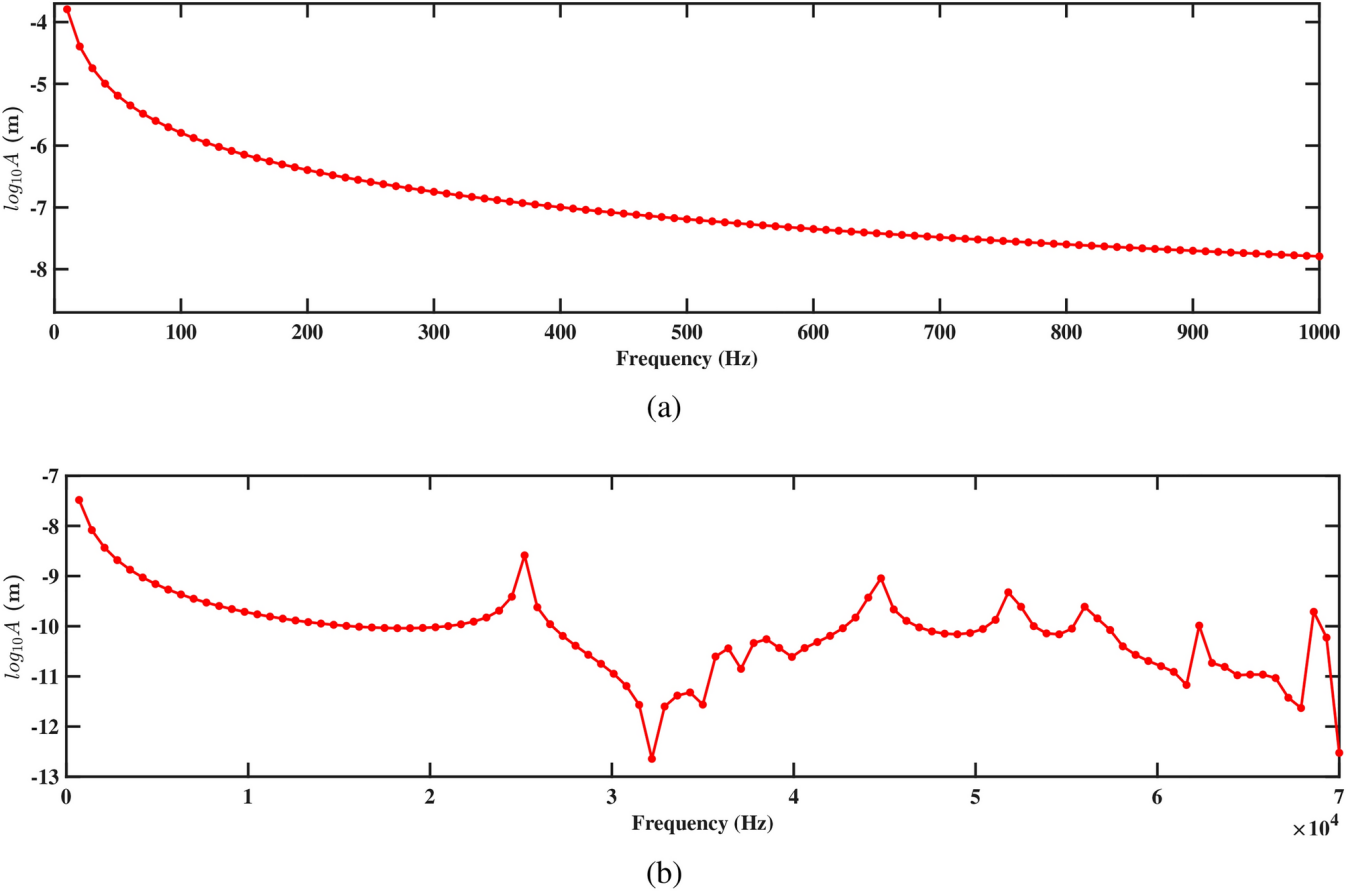
Amplitude with respect to the frequency corresponding to structure 2 (**a**) up to 1000 Hz, and (**b**) up to 70, 000 Hz, with a spacing distance of $d_2=0.0505$ m.

The frequency-amplitude response indicates that as frequency increases, the amplitude (in meters) decreases significantly, especially at higher frequencies. At lower frequencies (up to 1000 Hz), fluid-structure interaction is weak, causing a steady decline in amplitude due to inertial effects (see Figs. 23a and 24a). The fluid-conveying system introduces hydrodynamic damping, further reducing vibration amplitude. At high frequencies, particularly at 70, 000 Hz, the amplitude drops sharply, highlighting high-frequency damping from viscous forces in the fluid and material damping from the PZT patches (see Figs. 23b and 24b). In these cases, hydrodynamic forces interact more effectively with the beam’s motion, leading to increased energy dissipation and reduced amplitudes. This aligns with findings on high-frequency vibrations in fluid-conveying structures, where fluid damping and wave scattering result in rapid energy dissipation^31^. The short wavelength of high-frequency oscillations enhances wave interactions with the fluid, amplifying energy loss and decreasing vibration amplitude (see Figs. 23b and 24b).

**Figure 25:**
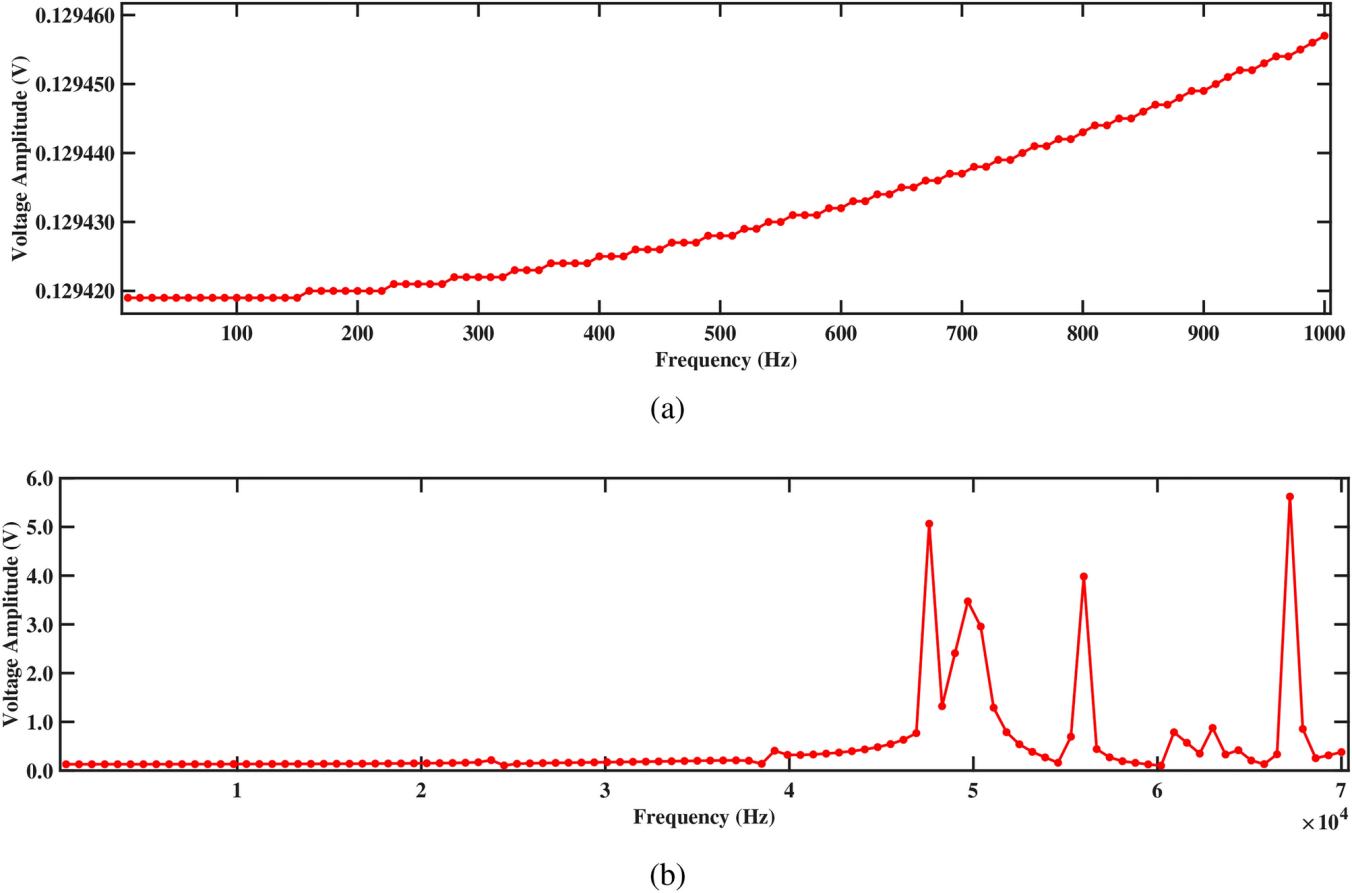
Voltage amplitude with respect to the frequency corresponding to structure 1 (**a**) up to 1000 Hz, and (**b**) up to 70, 000 Hz, with a spacing distance of $d_2=0.0505$ m.

**Figure 26:**
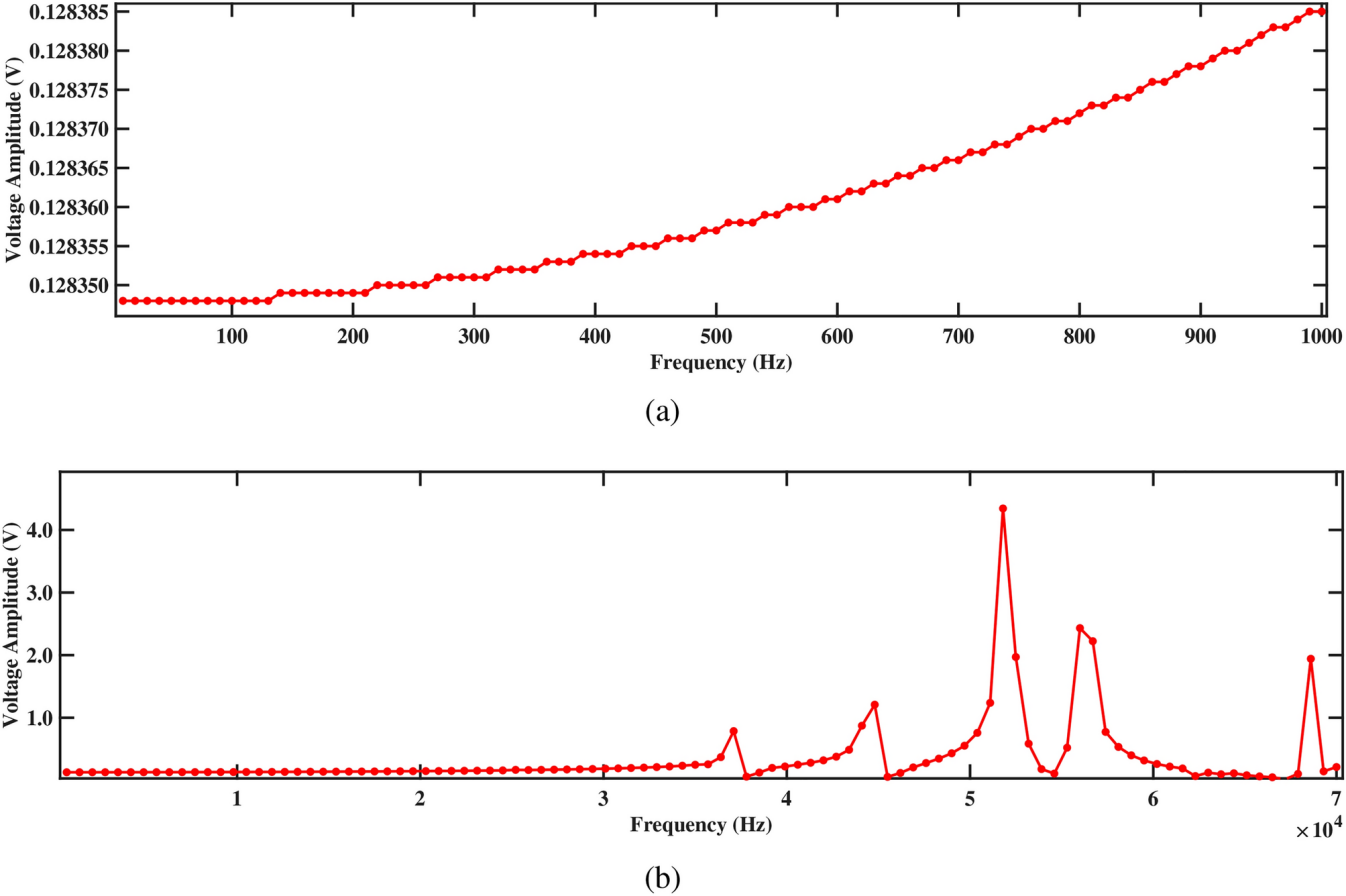
Voltage amplitude with respect to the frequency corresponding to structure 2 (**a**) up to 1000 Hz, and (**b**) up to 70, 000 Hz, with a spacing distance of $d_2=0.0505$ m.

Figures 25a and 26a illustrate the voltage variations for structure 1 and structure 2, respectively, across the frequency range of 0 to 1000 Hz. At a frequency of 1000 Hz, both structures exhibit minimal differences in voltage output, with structure 1 generating 0.12945 V and structure 2 producing 0.12838 V. This small difference is due to the low excitation frequency, where the fluid-structure interaction induces limited strain energy. Furthermore, at 70, 000 Hz, their performance improves significantly, with structure 1 reaching 5.62100 V and structure 2 generating 4.34333 V, as shown in Figs. 25b and 26b. At this higher frequency, the mechanical resonance amplifies oscillations, increasing strain and stress on the piezoelectric material. The enhanced energy transfer, combined with reduced damping, allows for a more efficient conversion of mechanical strain into electrical energy, maximizing the voltage output and improving energy harvesting efficiency.

The results clearly indicate that the structural spacing significantly influences both the natural frequencies and the voltage output of the system. The reason behind this is that the natural frequency of a structure is fundamentally determined by its stiffness and mass. In the present study, two structures 1 and 2 are mounted inside the circular pipe conveying fluid. The structures are fully submerged in the fluid, so additional factors came into play due to the fluid-structure interactions. Added mass effect is one significant factor where the surrounding fluids add mass to the structure. In this system, two beams are separated by a spacing distance $d_2$ which becomes more complex. This proximity of the structures influences the flow patterns and pressure distribution of the fluid, leading to changes in the effective stiffness and added mass. Cha et al.^32^ described the changes in resonance frequency of the structural vibrations due to the variations in the submersion length of the piezoelectric structure. The presence of the encompassing fluid is responsible for this effect by increasing the overall mass of the structure through the added mass effect and influencing its damping through the fluid-induced dissipation. Muthalif et al.^33^ noted the similar observation regarding this effect. Consequently, the natural frequencies are influenced by the spacing distance between the two structures. At the closest spacing of $d_2=0.0505$ m, strong mechanical coupling between the structures leads to lower natural frequencies and moderate voltage output. As the distance increases to $d_2=0.101$ m, the coupling weakens, resulting in slightly higher frequencies but a noticeable reduction in voltage, particularly for structure 2. The largest spacing, $d_2=0.202$ m, yields the highest natural frequencies and the best voltage performance for both structures, with significantly improved energy harvesting efficiency. This optimal separation reduces mechanical damping and interference, allowing the structures to resonate more independently and maximize energy transfer to the piezoelectric patches.

## Conclusions

The present study deals with the hydrodynamics and performance of the piezoelectric PZT-5A patches in the context of turbulent fluid flow. The numerical three-dimensional model consists of piezoelectric PZT-5A patches affixed to the beam and positioned inside an uniform circular pipe. The simulation is conducted for the turbulent flow conditions by directing fluid at an average velocity of 1.00 m/s towards two cylindrical structures placed in sequence. The first structure is positioned at 0.202 m downstream from the inlet of the circular pipe, while the second structure is located an equal distance downstream from the first beam. This arrangement is carefully designed to ensure optimal exposure of the structures to turbulent fluid flow. The beams are strategically placed perpendicular to the direction of fluid flow, allowing a comprehensive analysis of the fluid-structure interactions. To accurately model the turbulent flow and its effects, a sophisticated turbulent-flow model is utilized. This model effectively captures the complex fluid dynamics and interactions with the cylindrical structures. Finite element analysis (FEA) is conducted using ANSYS 2022 R1 to ensure precise numerical computation and to provide a detailed visualization of the fluid-structure interactions. Through the study of critical parameters such as voltage output, total deformation, Von-Mises stress distribution, strain profiles, frequency response characteristics, phase response behavior, and amplitude variations, this work sheds light on the intricate dynamics governing the behavior of the system. These significant findings highlight how the system performs and responds under turbulent flow conditions.

Turbulent fluid flow offers significant advantages for voltage generation, highlighting increased coherence, a robust driving mechanism, and vortex-shedding phenomena. These factors contribute to a maximum voltage output of 0.41264 V at a frequency peak of 1000 Hz, showcasing its superior performance over laminar flow conditions. The voltage generated from the turbulent fluid flow within a pipe conveying fluids showcases its substantial potential for diverse applications. In industrial and environmental contexts, this voltage can drive actuators, enabling precise fluid flow manipulation for optimal process control, which is crucial for maintaining efficient pipeline operations. Additionally, the generated voltage can power sensing devices that offer real-time monitoring of fluid properties and flow dynamics, thus enhancing process control and optimization efforts within the fluid conveyance systems. In biomedical engineering, this voltage can power implantable medical devices for therapeutic or diagnostic purposes, presenting innovative healthcare solutions. On a micro-scale, the voltage is practical for charging cell phones, illuminating LED lights, and powering bulbs, especially in areas lacking conventional power sources^34^. Storing this voltage in batteries ensures a sustainable power supply for future use. In general, the voltage obtained from turbulent fluid flow within pipes not only boosts the efficiency of fluid systems but also provides a versatile and reliable power source for a broad range of applications. Furthermore, the optimal configuration for efficient energy harvesting is achieved with a spacing of $d_2=0.202$ m. This configuration allows both structures to operate with minimal interference, leading to higher natural frequencies and improved energy transfer. The reduced mechanical interaction ensures more effective strain distribution across both structures, resulting in significantly higher voltage outputs at both low and high frequencies. The larger spacing enhances independent resonance, reduces damping, and maximizes energy harvesting potential, making $d_2=0.202$ m the most efficient spacing for this system.

The findings of this study open new avenues for future research on optimizing PEHs in turbulent flow conditions within pipe systems. In this study, a series configuration of beams with PZT patches is used, and the results suggest that future research could look into the benefits of different PEHs, such as tandem PEHs, by adjusting water speed and spacing ratios to further improve energy harvesting efficiency. From the work of Yadav et al.^29,35^ on turbulent and laminar flow for a single beam, it is evident that there is potential for even greater energy generation through more complex configurations. Future efforts could refine the PEHs design and configuration to maximize energy extraction efficiency under varied flow conditions and environmental factors within pipes. Additionally, significant improvements in energy generation in high-speed flow regions indicate the feasibility of developing multi-piezoelectric harvester systems arranged in parallel to boost energy production. This study provides a solid foundation for future advancements in energy harvesting from water flow in pipes, paving the way for sustainable energy solutions.

## Data Availability

The datasets used and/or analysed during the current study are available from the corresponding author on reasonable request.

## List of symbols

$$\mu$$: Dynamic viscosity
$$\mu _k$$: Turbulent viscosity
$\epsilon$: Turbulent dissipation rate
$$P_k$$: Production term
$\varvec{n}$: Normal component of the unit vector
$C_\mu$: Model constant used in turbulence models
$$\tau$$: Shear stress
$\varvec{u}_{tang}$: Shear velocity of the wall
$u+$: Velocity normal to the wall
$\delta ^+_w$: Separation distance from the wall
$$u_\tau$$: Friction velocity
$U_0$: Average velocity of flowing fluid
$$U_{ref}$$: Reference velocity
$$I_T$$: Turbulent intensity
$L_T$: Turbulent length scale
$\text {k}$: Von Karman constant
$p_0$: Discharge flow pressure
$$\widehat{p_0}$$: Reference pressure
$p_0$: Discharge flow pressure
$$U_s$$: Stream velocity
$$R_I$$: Pipe radius
*Re*: Reynolds number
*St*: Strouhal number
$f_S$: Vortex shedding frequency
*e*: Piezoelectric stress coefficient
$\epsilon ^S$: Constant strain of dielectric matrix
*D*: Stiffness matrix at constant electric field
$$C_d$$: Drag coefficient
$$C_i$$: Inertial coefficient
$$\varvec{F_i}$$: Inline force
$$V_p$$: Voltage
*A*: Area of the patch
$$\epsilon _T$$: Dielectric permittivity
$t_p$: Thickness of the piezoelectric material
$$K_f$$: Coupling factor
$d_c$: Charge coefficient for piezo-patch
*P*: Power produced by the piezo-patch
$R_p$: Resistance offered by the piezo-patch
*E*: Harvested energy
*t*: Duration of the energy harvesting process
PZT: Lead zirconate titanate
FIV: Flow-induced vibration
VIV: Vortex-induced vibration
PEHs: Piezoelectric energy harvesters
TKE: Turbulent kinetic energy
GPa: Gigapascal
nF: Nanofarad
